# Effects of *Escherichia coli* on Mixotrophic Growth of *Chlorella minutissima* and Production of Biofuel Precursors

**DOI:** 10.1371/journal.pone.0096807

**Published:** 2014-05-07

**Authors:** Brendan T. Higgins, Jean S. VanderGheynst

**Affiliations:** Biological and Agricultural Engineering, University of California Davis, Davis, California, United States of America; Oak Ridge National Laboratory, United States of America

## Abstract

*Chlorella minutissima* was co-cultured with *Escherichia coli* in airlift reactors under mixotrophic conditions (glucose, glycerol, and acetate substrates) to determine possible effects of bacterial contamination on algal biofuel production. It was hypothesized that *E. coli* would compete with *C. minutissima* for nutrients, displacing algal biomass. However, *C. minutissima* grew more rapidly and to higher densities in the presence of *E. coli*, suggesting a symbiotic relationship between the organisms. At an initial 1% substrate concentration, the co-culture produced 200-587% more algal biomass than the axenic *C. minutissima* cultures. Co-cultures grown on 1% substrate consumed 23–737% more of the available carbon substrate than the sum of substrate consumed by *E. coli* and *C. minutissima* alone. At 1% substrate, total lipid and starch productivity were elevated in co-cultures compared to axenic cultures indicating that bacterial contamination was not detrimental to the production of biofuel precursors in this specific case. Bio-fouling of the reactors observed in co-cultures and acid formation in all mixotrophic cultures, however, could present challenges for scale-up.

## Introduction

Biofuel production from microalgae has a number of advantages over biofuel from food crops. Microalgae grow rapidly, have lower land-use impacts than food crops, and can utilize saltwater and wastewater resources [1]. Furthermore, certain genera of algae, such as *Chlorella*, have the potential to accumulate lipids and starch, which can be converted to biofuel using existing technology [2]. In addition, many *Chlorella* species have the ability to utilize organic carbon sources, allowing microalgae to act as both a fuel production and conversion platform.

Studies have shown that increasing lipid productivity is the fastest way to achieve cost-effective liquid fuels from microalgae [3], [4]. Mixotrophic growth of microalgae (where both organic and inorganic carbon are utilized) has recently gained attention due to the high lipid productivities achieved using this cultivation platform. Numerous researchers have studied algae growth on glucose, glycerol, and acetate and concluded that mixotrophic growth can enhance lipid production by an order of magnitude [5]–[7].

Still, mixotrophic growth requires supplementation with organic carbon, which can have negative impacts on costs, the food supply, and the environment. In light of these challenges, studies of algae cultivated on organic-rich wastewaters have proliferated in the literature. Theoretically, algae can simultaneously treat the wastewater (removing nutrients) and produce biofuel precursors, thereby reducing cost and environmental impacts [8].

A major challenge with this concept is that other organisms may out-compete algae in nutrient-rich waters. Even if photobioreactors are utilized, contamination is still a concern and can lead to costly system shut-downs if not properly managed. To date, the effect of microbial contamination in mixotrophic cultures is poorly understood. Multiple studies of mixotrophic growth have been conducted using pre-sterilized wastewater (by autoclaving or sterile filtration) prior to inoculating with an algae monoculture [9]–[11]. These studies reported that organic-rich wastewater increased algae growth rates, and in some cases, lipid content as well. However, these results do not provide insight into how such a system would work in the presence of heterotrophic organisms. Given the presence of organic carbon, heterotrophic organisms, such as bacteria are expected to proliferate in mixotrophic cultures, particularly at high organic carbon concentrations. Such an event could drastically change the projections reported in the literature thus far on mixotrophic algae growth.

Limited research has been conducted on non-axenic algae cultures grown under mixotrophic conditions. Woertz et al. [12] cultivated a mixed algae culture on unsterilized municipal wastewater and diluted anaerobic digester effluent. Their research goal was primarily to study nitrogen and phosphorous removal so it was unclear if these cultures experienced significant mixotrophy. Their results indicated that simultaneous wastewater treatment and biofuel production is feasible, however, no results were presented on the role played by non-algae species in the cultures.

de-Bashan et al. [13] studied the effect of the growth-promoting bacterium, *Azospirillum brasilense,* on three algal species for the purpose of advanced wastewater treatment. They found that *Azospirillum* increased algae growth, lipid content, and fatty acid diversity, potential benefits for biofuel production. *A. brasilense's* growth promotion appeared to stem from the release of the plant hormone indole-3-acetic acid (IAA) [14]. These researchers established co-cultures within alginate beads and used synthetic wastewater whose organic carbon content was unspecified. In addition to *A. brasilense*, Lebsky et al. [15] co-cultured the bacterium *Phyllobacterium myrsinacearum* with *Chlorella vulgaris* in alginate beads in order to elucidate interactions between these species. They found that *P. myrsinacearum* showed no growth enhancing properties compared to *A. brasilense*.

Given limited prior research on mixotrophic algae growth, several lab-scale experiments were conducted in order to address the following question: What effect does bacterial contamination have on mixotrophic algal biomass, lipid, and starch production? Substrate utilization efficiency was also determined on the basis of substrate energy sequestered in lipids and starch. No studies have been identified that address the issue of substrate utilization efficiency, a critical parameter if non-waste carbon sources are used. *Chlorella minutissima* (UTEX 2341) was chosen due to its ability to accumulate lipids [16], [17] and utilize a variety of organic compounds. Defined media and controlled “contamination” of the algae culture with a single strain of *E. coli* were used in order to elucidate interactions that may emerge. *E. coli* was chosen because it grows rapidly under the culture conditions tested, competes with *Chlorella* for carbon substrates but is not known to be pathogenic toward algae. It can also be enumerated through plating, and is found in many wastewaters [18]. The carbon sources tested included glucose, glycerol and acetate, all of which enhanced growth in *Chlorella vulgaris* [5]. Glucose and glycerol enter metabolism through glycolysis whereas acetate enters via the TCA cycle, providing an opportunity to observe contrasts in biomass composition. The knowledge gained from these experiments can then be applied toward the study of microbial communities and wastewater of increasing complexity.

## Methods and Materials

### Algae cultivation

*Chlorella minutissima* (UTEX 2341) pre-cultures were initiated from selected colonies on ATCC No. 5 agar medium [19]. Pre-cultures were grown in N8-NH_4_ medium (supplemental material), adjusted to pH 7.2, until a cell density of 10^7^ cells per ml was reached (about 7–8 days). Cells were counted with a hemocytometer. The pre-cultures were grown in 1 L bottles filled to 800 ml and aerated with 2% (v/v) CO_2_ in air at 400 ml per minute under 10,000 lux illumination (16∶8 light-dark cycle). Cultures were mixed by magnetic stir bar (∼300 rpm) and maintained at ∼28°C.

Algae cells in the pre-culture were concentrated by overnight settling before inoculation into autoclaved N8-NH_4_ media in 250 ml hybridization tubes (filled to 200 ml) to achieve 10^7^ cells/ml. Glucose, glycerol, or sodium acetate (pH adjusted to 7.2 with HCl) stock solutions were sterile filtered (0.2 µm) and added to achieve the desired substrate concentration (0, 0.5, 2, and 10 g/L). Each substrate type was tested in sequential batches along with autotrophic control cultures. All three substrates were tested with axenic *C. minutissima*, axenic *E. coli*, and co-culture conditions for a total of nine batch runs, each of which ran for five days.

Stock *Escherichia coli* (ATCC 25922) were cultivated on liquid LB medium with 1% glucose for ∼24 hours and then inoculated into the reactors to achieve ∼10^4^ CFU/ml. This value was chosen because it falls in the range of coliform bacteria concentration found in treated municipal wastewaters (without disinfection). Zhang and Farahbakhsh [20] observed coliform concentrations of 10^3^–10^5^ CFU/ml in secondary wastewater. Guardabassi et al. [21] measured coliform concentrations of 10^3^–10^5^ CFU/ml in tertiary wastewater and total bacteria concentrations in the range of 10^5^–10^6^ CFU/ml.

Axenic algae cultures were supplied with an initial dose of kanamycin at 20 mg/L in order to reduce the risk of contamination in the hybrid tubes. Initial testing showed that kanamycin had no detectable impact on the growth of *C. minutissima*. The hybrid tube reactors were placed in a water bath maintained at 28°C under 10,000 lux illumination (16∶8 light-dark cycle). Stir bars were used to sweep the bottom of the reactors (∼100 rpm) with aeration tubes providing 125 ml per minute of ambient air. CO_2_ supplementation was not provided to the hybrid tubes because research has suggested that excess carbon dioxide can inhibit organic carbon uptake in mixotrophic cultures [22]. Optical density (OD_550_) was measured each day in a microplate reader (Spectramax M2, Molecular Devices, Sunnyvale, CA) and correlated to total dry weight at the end of each five-day culture period. Unique correlations were developed for each substrate and culture condition.

### Biomass composition

Biomass was harvested at the end of the five-day culture period by centrifugation at 5000 g for 5 minutes (IEC MultiRF, Thermo Electron Corp., Waltham, MA) and the pellet was washed three times with dH_2_O to remove media salts. The pellet was lyophilized at −45°C (Freezone4.5, Labconco, Kansas City, MO) and weighed. Dry biomass was suspended in Folch solvent (2∶1 chloroform and methanol) and cells were lysed with 0.5 mm zirconia-silica beads with a bead beater (FastPrep FP120, Savant Instruments, Holbrook, NY). Beating was performed for six 20-second periods at maximum speed with samples cooled on ice between periods. Beads were removed with a mesh filter and washed with Folch solvent. NaCl solution (0.9%) was added to the disrupted cell suspension to achieve a phase separation between the polar and non-polar fractions. The non-polar (chloroform) fraction was removed and stored at −20°C for later assay with the sulfa-vanillin-phosphoric acid (SPV) assay. The SPV assay has been shown to correlate well with gravimetric lipid found in *Chlorella* [23]. Corn oil was used as the assay standard. Select crude lipid extracts were dried down and weighed. The assay was found to slightly underestimate gravimetric crude lipid.

The polar fraction was washed three times with acetone and then three times with water by centrifugation at 12,000 g for 5 minutes followed by supernatant removal. The resulting pellet was freeze-dried, ground with a micropestle, and the starch was gelatinized by heating the pellet in dH_2_O for 30 minutes at 80°C. The gelatinized starch suspension was hydrolyzed with α-amylase (7.5 U/ml) and α-amyloglucosidase (3 U/ml) in acetate buffer (pH 5) overnight at 37°C. The suspension was centrifuged and the supernatant removed for analysis. Dinitrosalicylic acid (DNS) was used to detect reducing sugars formed in the enzyme-substrate reaction with glucose standards [24]. Starch mass was determined by multiplying the sample glucose content by 0.9 to correct for water added during starch hydrolysis.

### HPLC analysis

Daily culture samples were collected over the five-day growth period, centrifuged at 15,000 g to remove cells, and filtered at 0.2 µm (Titan2 PTFE, SUN-SRi, Rockwood, TN). High performance liquid chromatography (HPLC) was used to quantify organic compounds in the media (Prominence Liquid Chromatograph, Shimadzu Corp., Kyoto, Japan). An Aminex 87H column (BioRad, Hercules, CA) was used to separate glucose, glycerol, acetate, and secreted organic compounds in the culture media. The column oven was set to 60°C and 5 mM sulfuric acid in Milli-Q water was used as the mobile phase under isocratic conditions (0.6 ml/min). Refractive index and UV detectors were used to quantify substrate concentrations.

### E. coli enumeration

Viable *E. coli* were determined by plating on LB agar medium with 1% glucose. Plating was performed at 0, 24, 48, and 96 hours for cultures containing *E. coli*. Quantitative PCR of 16S rDNA was used to determine total bacterial biomass in the final freeze-dried co-culture samples. A similar approach has been employed in the quantitation of bacterial populations in a variety of environments [25], [26]. Freeze-dried material was re-suspended in dH_2_O and DNA was extracted using a FastDNA Spin Kit (MP Biomedicals, Solon, OH) according to the manufacturer's instructions. A 110 base portion of *E. coli* 16S rDNA was amplified using the forward primer 5′-CAAGACCAAAGAGGGGGACC-3′ and reverse primer 5′-TCAGACCAGCTAGGGATCGT-3′ (Invitrogen Life Technologies, Grand Island, NY). SYBR Green PCR Master Mix (Applied Biosystems, Warrington, UK) was used in a StepOnePlus qPCR instrument (Applied Biosystems, Foster City, CA). The program ran for thirty cycles: 95°C for 15 sec, 60°C for 15 sec, 72°C for 30 sec. Gel electrophoresis was used to confirm correct amplification. Previously amplified PCR product from *E. coli* was used as a DNA standard. Samples of pure *E. coli* cultured under similar conditions to the co-cultures were used to correlate *E. coli* dry weight to 16S rDNA concentration. Double stranded DNA concentrations were measured using a Qubit assay kit (Invitrogen, Eugene, OR).

### Substrate conversion efficiency

Efficiency of substrate utilization was determined on an energy basis (Equation 1):
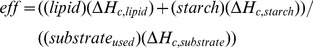
(Equation 1)



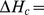
standard enthalpy of combustion. Enthalpy of combustion for lipids and starch were assumed to be 37 and 15.7 kJ/g, respectively [27]. Glucose, glycerol, and acetate were assumed to contain 15.6, 15.6, and 14.6 kJ/g, respectively [27], [28]. The rationale for this method is that the substrate could be used for direct combustion or it could be converted to biofuel precursors by algae, thereby losing useable energy in the conversion process.

### Data analysis

JMP v.9 software (SAS Institute, Cary, NC) was used to conduct ANOVA and Tukey tests on sample means. These statistical tests require homogeneity of variance between data groups, however, certain measurements exhibited variances that increased with the sample mean. Power transformation of data prior to statistical analysis is the recommended course of action in order to ensure that basic assumptions such as data normality and homogeneity of variance are not violated [29], [30]. To correct heteroscedasticity, data were transformed prior to statistical analyses using power transforms based on procedures developed by Box and Cox [31], [32]. The form of the Box-Cox transform is expressed in Equation 2 where lambda was optimized using JMP to minimize heteroscedasticity in the transformed data (y′).



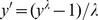
(Equation 2)


In the majority of cases, lambda was found to be close to zero, in which case the data were transformed by taking the natural logarithm. Prior to further statistical analyses, all data were visually inspected for heteroscedasticity and checked with Levene's test to ensure non-significance. Significance for all tests was assessed at the 0.05 level.

## Results

### Biomass productivity

**Figure 1:**
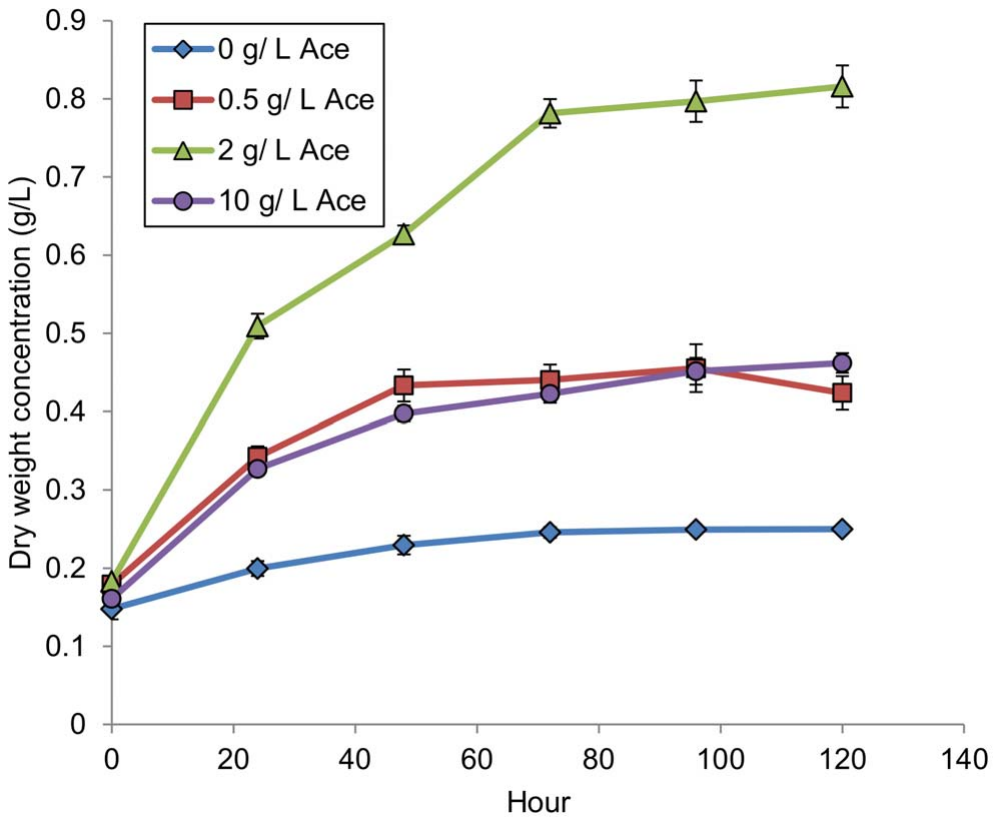
Growth curve for axenic acetate cultures. Error bars are standard deviations based on three biological replicates.

Substrate concentration had a large impact on total biomass and lipid productivity in axenic algae cultures (Table 1). Increasing substrate concentration from 0.5 g/L to 2 g/L consistently yielded a 1.6 fold increase in biomass productivity. Productivity in glucose and glycerol cultures exhibited saturation behavior with increasing substrate concentration: cultures supplied with 10 g/L of substrate did not produce proportionally more biomass than 2 g/L substrate. In contrast, the axenic acetate cultures exhibited apparent substrate inhibition where the 10 g/L cultures produced significantly less biomass than the 2 g/L cultures (p = 0.002, Tukey test). The initial growth rate (first 24 hours) in the 10 g/L acetate culture was about half that observed in the 2 g/L culture (Figure 1). Growth curves for glucose and glycerol can be found in Figures S1 and S2.

**Table 1:** Productivity levels and lipid and starch contents for axenic *C. minutissima* (top) and co-cultures of *C. minutissima* and *E. coli* (bottom).

Culture conditions	Biomass productivity (mg/L/d)	Lipid productivity (mg/L/d)	Starch productivity (mg/L/d)	Final lipid content (%)	Final starch content (%)
Axenic *C. minutissima*					
Glucose					
0 g/L	8.4 (1.1) c	2.0 (0.2) b	0.09 (0.01) c	23.5 (2.4) a	1.04 (0.06) bc
0.5 g/L	38.0 (2.4) b	5.1 (1.4) b	0.30 (0.04) c	13.4 (2.8) b	0.78 (0.06) c
2 g/L	98.8 (4.9) a	14.5 (1.3) a	1.86 (0.68) b	14.7 (0.6) b	1.90 (0.68) b
10 g/L	98.7 (6.0) a	13.5 (1.8) a	3.16 (0.34) a	13.7 (1.1) b	3.19 (0.34) a
Glycerol					
0 g/L	13.5 (0.8) d	2.8 (0.1) d	0.27 (0.01) c	20.8 (0.5) a	1.98 (0.15) c
0.5 g/L	42.3 (1.5) c	7.5 (0.4) c	0.66 (0.04) c	17.8 (1.5) a	1.56 (0.05) c
2 g/L	111.0 (0.8) b	19.6 (1.5) b	4.04 (1.01) b	17.7 (1.4) a	3.65 (0.93) b
10 g/L	123.4 (0.9) a	23.3 (1.8) a	7.75 (0.78) a	18.9 (1.6) a	6.28 (0.67) a
Acetate					
0 g/L	19.9 (3.4) d	3.3 (0.6) d	0.20 (0.03) d	16.6 (0.3) a	1.03 (0.06) b
0.5 g/L	48.3 (2.3) c	6.7 (0.6) c	0.42 (0.02) c	13.8 (0.6) b	0.87 (0.02) c
2 g/L	125.8 (2.9) a	18.5 (0.7) a	0.84 (0.04) b	14.7 (0.2) b	0.67 (0.02) d
10 g/L	59.6 (3.9) b	8.8 (0.8) b	0.98 (0.03) a	14.8 (1.3) b	1.65 (0.06) a
*C. minutissima, E. coli*					
Glucose					
0 g/L	10.5 (1.8) d	2.1 (0.3) d	0.16 (0.02) d	20.2 (0.4) b	1.51 (0.04) bc
0.5 g/L	85.0 (3.5) c	14.5 (1.1) c	1.12 (0.05) c	17.0 (0.9) c	1.31 (0.02) c
2 g/L	190.8 (14.8) b	25.9 (3.5) b	3.48 (0.54) b	13.6 (1.0) d	1.67 (0.04) b
10 g/L	342.8 (65.6) a	83.9 (15.8) a	59.28 (9.70) a	24.5 (1.4) a	17.43 (2.11) a
Glycerol					
0 g/L	12.9 (4.9) d	1.5 (0.5) c	1.25 (0.50) c	12.0 (1.0) b	9.71 (0.58) b
0.5 g/L	74.7 (5.3) c	9.5 (1.8) b	3.79 (0.40) b	12.6 (1.6) b	5.07 (0.32) c
2 g/L	172.4 (15.5) b	22.0 (2.5) b	5.78 (2.50) b	12.2 (0.5) b	4.00 (0.11) c
10 g/L	419.8 (29.6) a	72.2 (8.4) a	76.97 (8.13) a	17.2 (0.8) a	18.31 (0.65) a
Acetate					
0 g/L	34.6 (2.5) d	6.0 (0.1) d	0.97 (0.06) b	17.2 (0.8) a	2.83 (0.36) b
0.5 g/L	87.5 (4.3) c	13.4 (1.3) c	1.06 (0.03) b	15.3 (0.8) a	1.22 (0.06) c
2 g/L	207.7 (1.4) b	26.6 (1.1) b	0.76 (0.11) c	12.9 (0.5) b	0.39 (0.05) d
10 g/L	428.9 (6.3) a	72.5 (4.7) a	26.52 (3.44) a	16.9 (0.8) a	6.18 (0.78) a

Productivities are five-day averages and lipid and starch contents are a percent of dry weight. Three biological replicates were used in all cases except for co-cultures grown on 10 g/L glycerol where only two replicates were used. Mean value is followed by standard deviation in parenthesis. Within substrate batches (e.g. axenic *C. minutissima* w/glucose), values followed by the same letter are not significantly different at the 0.05 level.

In light of saturation behavior, attempts were made to fit biomass productivity to a Monod model based on substrate concentration, however, results only fit for the first 24 hours of growth. Thereafter, growth rates were lower than the model predicted, suggesting that other factors besides substrate concentration had an effect on productivity. Mean comparisons among substrate types were not performed due to the batch effect apparent in autotrophic control cultures. With the exception of 10 g/L acetate, productivity differences among the three substrates (holding concentration constant) were typically within 20% of each other and are unlikely to be of practical significance.

**Figure 2:**
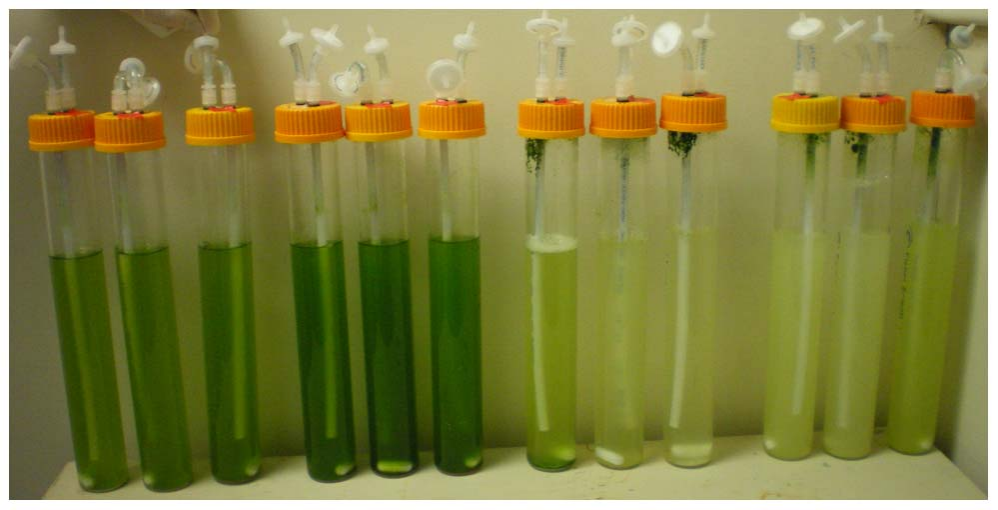
Bio-fouling in glucose co-cultures. From left to right in triplicate: 0 g/L glucose, 0.5 g/L glucose, 2 g/L glucose, 10 g/L glucose. Similar fouling occurred in glycerol and acetate co-cultures. A film of algae can be seen on the upper wall of reactors under high glucose concentrations.

Co-cultures, which contained *C. minutissima* and *E. coli*, had higher biomass, lipid, and starch productivity than axenic *C. minutissima* cultures (Table 1). At 10 g/L substrate, total biomass productivities were 240% to 619% higher for co-cultures compared to axenic cultures which was significantly higher than the changes observed in autotrophic control cultures (p<0.0001). Likewise, lipid production was enhanced by 210–722% in these same cultures and the change was also significantly greater than that observed in autotrophic cultures (p<0.0001). Greater growth variability was observed in the glucose and glycerol co-cultures than in the axenic cultures. One possible explanation is the bio-fouling that occurred in all co-cultures under high substrate concentrations (Figure 2). Biomass was scraped from the walls of the reactors and re-suspended on a daily basis prior to sampling. Significant bio-fouling did not occur until day three of cultivation (late log phase) and appeared to result from media froth caused by *E. coli*. The rising bubbles in the reactor did not break at the liquid interface, allowing them to lift cells out of suspension. In addition, the algae appeared to coagulate and stick to the wall of the reactor. Antifoam was not used to overcome this challenge since the use of such products in large-scale cultivation is unlikely due to cost.

### pH fluctuations in mixotrophic cultures

**Figure 3:**
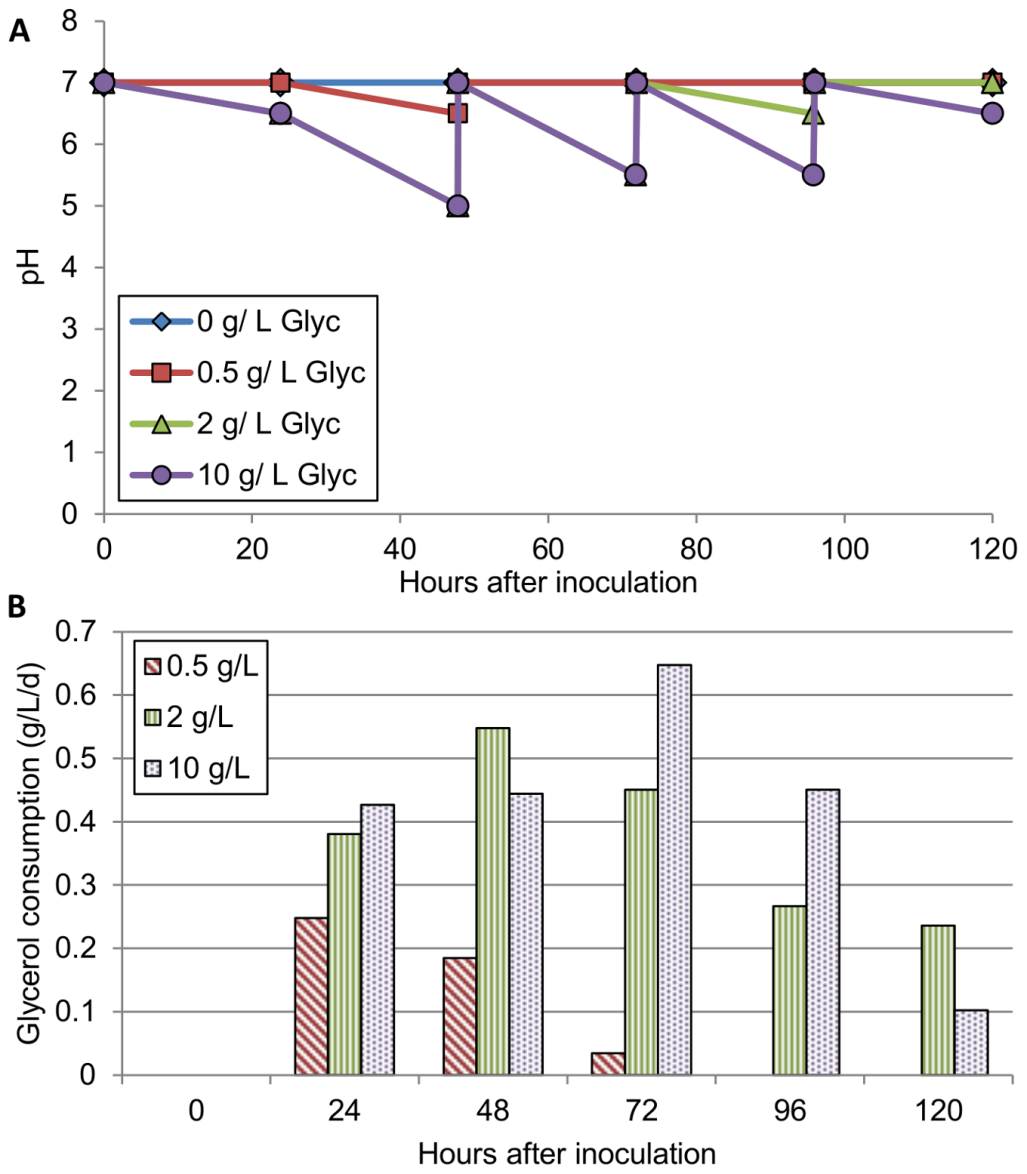
pH change and glycerol consumption in axenic *C. minutissima* cultures. A: The line graph shows a decline in pH. Similar pH declines were observed in glucose cultures. pH was re-adjusted to neutral conditions every 24 hours. B: The bar graph shows the rate of glycerol consumption by the cultures during each 24-hour period.

The autotrophic (no carbon substrate) cultures had no detectable pH change over the five-day growth period and did not require pH adjustment. In cultures supplied with glucose and glycerol, pH decreased and required adjustment with 3 M NaOH on a daily basis to restore neutral conditions (Figure 3). Additional pH graphs can be found in Figures S3 and S4. The decline in pH appeared to coincide with substrate consumption as measured by HPLC (Figure 3). Once a substrate was depleted, the decline in pH ceased and no further pH adjustments were required. Similar pH declines were observed in cultures of axenic *C. minutissima*, axenic *E. coli*, and in co-cultures. Cultures grown on acetate experienced an increase in pH and required daily adjustment to pH 7 using 3 M HCl suggesting that cultures consumed acetate in its protonated form. Secretion of detectable formic and acetic acid was observed via HPLC in axenic *E. coli* cultures grown on glucose and glycerol. At 0.5, 2, and 10 g/L glucose, final formic acid concentrations were 0.014, 0.069, and 0.036 g/L, respectively. For the same cultures, final acetic acid levels were 0, 0.092, and 0.141 g/L, respectively. Neither compound was detected in co-cultures. Identification of compounds secreted by *C. minutissima* under mixotrophic growth is the subject of ongoing investigation but the present analysis showed that *C. minutissima* did not secrete typical anaerobic products such as lactate, formate, or acetate. Some work on secretions of *C. minutissima* grown on solid media has already been published, indicating that this algae secretes a complex mixture of lipids among other metabolites [33].

### Bacteria and algae levels in co-cultures

Colony counts of viable *E. coli* concentration in co-cultures increased rapidly on days one and two, then plateaued over the remainder of the culture period (Figures S5, S6, and S7). Order of magnitude differences in CFU concentration were observed in co-cultures for different initial substrate concentrations. Zero, 0.5, 2, and 10 g/L initial glucose concentrations resulted in CFU concentrations of 3.9×10^6^, 7.7×10^7^, 1.5×10^9^, and 1.5×10^9^ CFU/ml, respectively, after 96 hours (Table 2). This result indicates saturation of viable *E. coli* with respect to substrate concentration.

**Table 2:** Viable cell densities (CFU/mL) of *E. coli* at 96 hours for co-cultures (top) and axenic *E. coli* cultures (bottom).

		Initial substrate concentration			
Culture condition		0 g/L	0.5 g/L	2 g/L	10 g/L
*C. minutissima, E. coli*					
Glucose		3.9E+06	7.7E+07	1.5E+09	1.5E+09
Glycerol		7.4E+06	6.4E+07	3.3E+09	4.8E+09
Acetate		9.0E+06	1.5E+08	6.1E+08	1.5E+09
*E. coli*					
Glucose		7.0E+05	3.1E+07	5.0E+07	3.0E+07
Glycerol		-	6.3E+06	2.3E+07	3.0E+06
Acetate		-	1.4E+07	3.3E+07	2.4E+05

Because both non-viable and viable bacteria contributed to co-culture biomass, PCR amplification of 16S rDNA was used to quantify total *E. coli* biomass in the co-cultures (58 g *E. coli* dry weight/µg 16S rDNA). DNA extraction using a bacterial cell-lysing buffer appeared to extract DNA almost exclusively from bacteria (*Chlorella* extract contained 6.4 µg/ml DNA extract compared to 154 µg/ml extract for *E. coli*). The ratio of 16S rDNA to total extracted DNA was also roughly constant in co-cultures (6.8×10^−6^, 6.6×10^−6^, 6.6×10^−6^ µg 16S rDNA/µg extracted DNA for glucose, glycerol, and acetate batches respectively). Furthermore, the presence of algae in the samples appeared to have almost no impact on either DNA extraction or the PCR reaction. This was demonstrated using pre-mixed algae and *E. coli* cultures that were analyzed as a validation step. The mixtures contained 0%, 25%, 50%, 75%, and 100% *E. coli* by mass and PCR analysis resulted in calculated *E. coli* fractions of 0%, 24%, 46%, 69%, and 100%, respectively.

The qPCR results mirrored the CFU counts except at 10 g/L substrate. The qPCR results indicated that *E. coli* concentrations (g *E. coli* dry weight/L) were lower at 10 g/L substrate than at 2 g/L although this difference was only statistically significant for glucose. However, the percent of biomass as *E. coli* was significantly lower at 10 g/L substrate than at 2 g/L for all substrates (Table 3). This result was surprising given the hypothesis that *E. coli* were expected to occupy a larger fraction of culture biomass with increasing carbon substrate.

**Table 3:** Percent of dry biomass as *E. coli* in co-cultures based on qPCR and the percent increase of algae productivity in the co-culture versus the equivalent axenic culture (Table 1).

Substrate	Final *E. coli* content (%)	*E. coli* productivity (mg/L/d)	Algae productivity (mg/L/d)	Increase in algae productivity vs. axenic (%)
Glucose				
0 g/L	1.3 (0.4) c	0.13 (0.02) c	10.4 (1.8) c	25 (32) c
0.5 g/L	12.0 (2.4) b	10.25 (2.39) b	74.7 (2.6) b	97 (12) b
2 g/L	47.3 (9.6) a	91.23 (25.86) a	99.6 (11.3) b	1 (10) c
10 g/L	6.2 (2.5) b	22.06 (10.61) b	320.7 (57.0) a	224 (40) a
Glycerol				
0 g/L	1.0 (1.0) c	0.16 (0.21) c	12.8 (4.7) d	−6 (34) b
0.5 g/L	18.3 (1.6) ab	13.62 (0.42) b	61.0 (5.4) c	45 (18) b
2 g/L	30.0 (8.2) a	52.56 (18.98) a	119.8 (3.5) b	8 (3) b
10 g/L	11.5 (0.5) bc	48.36 (5.70) a	371.4 (23.9) a	200 (20) a
Acetate				
0 g/L	0.4 (0.2) c	0.13 (0.07) c	34.5 (2.5) d	75 (17) b
0.5 g/L	2.7 (0.3) b	2.35 (0.37) b	85.1 (4.0) c	76 (13) b
2 g/L	18.7 (3.2) a	38.71 (6.46) a	169.0 (7.9) b	34 (3) c
10 g/L	4.7 (0.9) b	20.06 (3.57) a	408.9 (7.9) a	587 (33) a

Productivities are five-day averages. Three biological replicates were used in all cases except for co-cultures grown on 10 g/L glycerol where only two replicates were used. Mean value is followed by standard deviation in parenthesis. Within substrate batches values followed by the same letter are not significantly different at the 0.05 level.

One possible explanation for lower apparent *E. coli* levels at higher substrate concentration is that DNA extraction was not representative of *E. coli* biomass in co-cultures. Presumably this would be the result of DNA leakage into the media due to cell lysis. To test this hypothesis, culture samples were tested for their DNA content after high-speed centrifugation (15,000g) and 0.2 µm filtration to remove cells (Titan2 PTFE, SUN-SRi, Rockwood, TN). Supernatants from cultures supplied with 0, 2, and 10 g/L glucose were found to contain 0.018, 2.47, and 0.296 µg/ml DNA extract, respectively. This suggests that leakage cannot explain the lower extracted DNA from 10 g/L cultures.

Based on the PCR result, algal biomass constituted 88.5–95.3% of total biomass in the co-cultures grown on 10 g/L substrate, indicating little displacement of algae by bacteria. More significantly, algal productivity in the co-cultures was 200–587% higher than algal productivity in axenic cultures at 10 g/L substrate suggesting a potential symbiotic relationship between the algae and bacteria. For all three substrates, these increases in algae productivity were significantly greater than differences observed in the autotrophic control cultures (p < 0.001 for all cases) indicating that symbiosis may have depended on the presence of organic substrates. Interestingly, the high bacterial fraction in the 2 g/L co-cultures coincided with only a minimal increase in algal productivity.

### Axenic E. coli cultures

To better understand *E. coli* response to substrate treatments, axenic *E. coli* cultures were grown on glucose, glycerol, and acetate under identical conditions to the co-cultures. *E. coli* productivity in axenic cultures was similar to *E. coli* productivity in co-cultures, lending credibility to the qPCR results (Table 4). Moreover, axenic *E. coli* cultures supplied with 10 g/L of glucose and acetate had significantly lower productivity (Table 4) than those supplied with 2 g/L glucose (p = 0.028) and 0.5 g/L acetate (p<0.001), respectively. In fact, axenic *E. coli* exhibited no growth at 10 g/L acetate compared to ∼22 mg/L/day at 0.5 g/L. Substrate inhibition is the most likely explanation for this observation since exponential growth was also delayed at 2 g/L acetate (began at 96 hours) compared to 0.5 g/L acetate which began at 24 hours (Figure S8). *E. coli* cultures grown on glucose and glycerol exhibited comparable initial growth rates across all substrate concentrations indicating minimal substrate inhibition. Elevated *E. coli* densities at 2 g/L glucose could be associated with the more favorable (less acidic) pH conditions that prevailed in the culture toward the end of the culture period (Figures S9 and S10 for growth and pH curves). However, a similar increase in growth did not take place in cultures grown on 2 g/L glycerol (Figure S11).

**Table 4:** Axenic *E. coli* growth measured gravimetrically.

Substrate	*E. coli* productivity (mg/L/d)	Final *E. coli* density (mg/L)
Glucose		
0 g/L	0.05 (0.91) c	1.5 (4.4) c
0.5 g/L	38.01 (0.53) b	194.5 (2.5) b
2 g/L	87.99 (17.91) a	439.1 (90.1) a
10 g/L	56.7 (0.80) b	288.3 (4.0) b
Glycerol		
0 g/L	-	-
0.5 g/L	32.90 (1.00) b	171.8 (4.5) b
2 g/L	62.44 (3.16) a	320.7 (14.7) a
10 g/L	63.09 (1.94) a	325.2 (11.9) a
Acetate		
0 g/L	-	-
0.5 g/L	22.12 (0.41) a	115.3 (1.9) a
2 g/L	40.04 (34.03)*	202.9 (170.3)*
10 g/L	1.27 (0.25) b	11.0 (1.6) b

Productivities are five-day averages. Mean values are followed by standard deviations in parenthesis. Within a batch, values connected by the same letter are not significantly different at the 0.05 level.

*Variability was too large to include this treatment in the multiple comparison.

Viable cell counts at nearly all time points for co-cultures were one to three orders of magnitude higher than those in axenic *E. coli* cultures (Table 2). As an example, viable cell counts at 96 hours at 10 g/L substrate were 51, 1600, and 6400 times higher in co-cultures than in axenic *E. coli* cultures when grown on glucose, glycerol, and acetate, respectively. This suggests that viable *E. coli* constituted a greater fraction of total *E. coli* biomass in co-cultures than in axenic cultures indicating *E. coli* may have benefitted from the presence of algae in co-cultures.

### Lipid and starch content

Among axenic *C. minutissima* cultures, the highest lipid contents were observed under autotrophic conditions (Table 1). For co-cultures, the treatments with 10 g/L glucose yielded the highest lipid contents with autotrophic cultures close behind. The high lipid content at 10 g/L substrate is further evidence that algae were the predominant species in these cultures. As expected, axenic *E. coli* cultures had low lipid content (3.4–5% when grown on glucose).

Total starch exhibited greater differences among culture conditions than lipid content (Table 1). The *E. coli* cultures grown on glucose yielded low glycogen levels (<1% of total biomass) that were barely detectable using the DNS assay. Therefore, it is unlikely that *E. coli* contributed significant glycogen to the elevated levels of α-glucan polymers observed in co-cultures. Axenic cultures grown on 10 g/L acetate produced 48 and 74% less starch than cultures grown on 10 g/L glucose and glycerol, respectively. These differences were significantly greater than those observed in the autotrophic control cultures from these respective batches (p<0.001, t-test). In all cases, 10 g/L of substrate resulted in the highest level of starch with very large increases observed in glucose and glycerol co-cultures (>15% starch content).

### Effect of kanamycin on biomass composition

Early testing showed that kanamycin had no measureable effect on growth rates of *C. minutissima* compared to no-antibiotic controls whereas tetracycline resulted in substantial growth inhibition (data not shown). A full factorial experiment was later conducted in which axenic *C. minutissima* was grown on 2 and 10 g/L glucose with and without kanamycin in order to determine possible effects on biomass composition. Kanamycin decreased biomass productivity by 9% (p = 0.56) and 13% (p = 0.054) in cultures supplied with 2 and 10 g/L glucose, respectively (Table 5).

**Table 5:** Productivity levels and starch and lipid contents for axenic *C. minutissima* cultures grown with and without kanamycin.

Culture conditions		Biomass productivity (mg/L/d)	Lipid productivity (mg/L/d)	Starch productivity (mg/L/d)	Final lipid content (%)	Final starch content (%)
2 g/L glucose	No kanamycin	117.2 (2.7) b	16.3 (1.3) ab	4.22 (1.93) c	13.9 (0.8) a	3.58 (1.57) c
	Kanamycin	106.0 (4.8) b	15.3 (2.6) b	2.56 (0.79) c	14.4 (2.2) a	2.40 (0.65) c
10 g/L glucose	No kanamycin	172.4 (2.6) a	23.8 (0.9) a	26.95 (3.07) a	13.8 (0.4) a	15.64 (1.77) a
	Kanamycin	148.4 (15.9) a	20.3 (4.6) ab	10.59 (0.80) b	13.6 (1.8) a	7.16 (0.30) b

Productivities are five-day averages. Mean values are followed by standard deviations in parenthesis based on three biological replicates. Within a column, values connected by the same letter are not significantly different at the 0.05 level.

Lipid content, as measured by the SPV assay, was nearly identical among treatments. In the 10 g/L glucose cultures, however, kanamycin appeared to significantly suppress starch levels. Two other possible explanations (culture contamination and assay interference) were tested and eliminated: all cultures were tested for bacterial contamination using 16S rDNA qPCR and had a negative result. Likewise, the glucose concentration from hydrolyzed starch was quantified using HPLC in addition to the DNS assay. Both methods showed that kanamycin cultures had about half the starch content as the no-kanamycin control. This difference was less pronounced for 2 g/L glucose.

### Substrate consumption

Substrate consumption levels (Table 6) appeared to mirror biomass productivity. Co-cultures consumed more substrate (and consumed it more rapidly) than axenic cultures when provided with 10 g/L substrate. When provided with 0.5 and 2 g/L substrate, axenic algae and co-cultures consumed all substrate within 5 days. Thereafter, growth slowed but did not cease suggesting continued autotrophic growth.

**Table 6:** Organic carbon substrate consumption (g/L) over a five-day growth period.

Initial substrate conditions	*C. minutissima*	*C. minutissima & E. coli*	*E. coli*
Glucose			
0.5 g/L	0.40 (0.01) b	0.47 (0.02) c	0.56 (0.01) c
2 g/L	1.70 (0.14) a	1.94 (0.02) b	2.09 (0.10) b
10 g/L	1.26 (0.29) a	6.09 (0.51) a	3.69 (0.56) a
Glycerol			
0.5 g/L	0.47 (0.00) b	0.50 (0.00) c	0.45 (0.02) b
2 g/L	1.88 (0.06) a	2.05 (0.04) b	1.94 (0.02) a
10 g/L	2.07 (0.07) a	6.26 (0.03) a	2.04 (0.26) a
Acetate			
0.5 g/L	0.46 (0.00) c	0.45 (0.00) c	0.46 (0.01) a
2 g/L	1.86 (0.04) a	1.91 (0.00) b	0.67 (0.58)*
10 g/L	0.96 (0.16) b	7.89 (0.20) a	0.00 (0.00) b

Mean values are followed by standard deviations in parenthesis based on three biological replicates (except for 10 g/L glycerol co-culture where n = 2). Within a batch, values connected by the same letter are not significantly different at the 0.05 level.

*Variability was too large to include this treatment in the multiple comparison.

Substrate uptake in the co-cultures was greater than the sum of substrate uptake by axenic *C. minutissima* and *E. coli* cultures. Specifically, the co-cultures consumed 23% more glucose, 59% more glycerol, and 737% more acetate than the sum of the axenic cultures. Axenic *E. coli* cultures consumed acetate at 0.5 and 2 g/L but did not consume any acetate when provided with 10 g/L, further indication of substrate inhibition. Likewise, axenic *C. minutissima* consumed only 0.96 g/L when grown on 10 g/L acetate yet the co-culture consumed 7.89 g/L, indicative of a symbiotic relationship that is linked to substrate uptake and utilization.

### Substrate conversion efficiency

**Figure 4:**
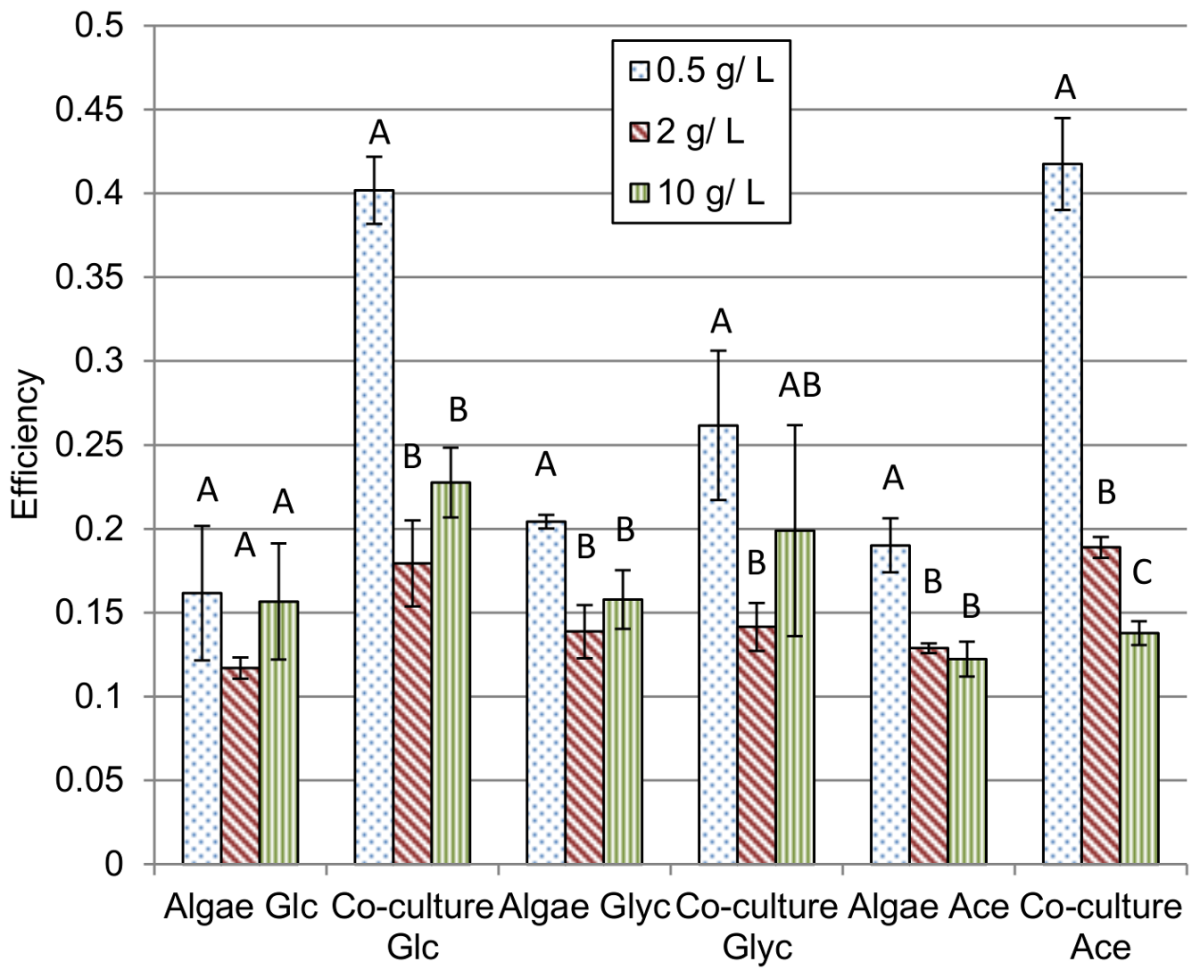
Efficiency of substrate conversion to biofuel precursors calculated using Equation 1. Glc  =  glucose, Glyc  =  glycerol, Ace  =  acetate. Energy obtained through photosynthesis is considered to be “free” so efficiency was not calculated for cultures with no substrate. Error bars are standard deviations based on 3 biological replicates with the exception of co-cultures grown on 10 g/L glycerol where n = 2. Within batches (same substrate and culture type), bars with the same letter are not significantly different.

Lower substrate concentrations generally led to greater substrate conversion efficiencies (Figure 4) with 0.5 g/L substrate supporting the highest efficiency. This is likely due to the significant contribution of photosynthesis to biomass growth in these cultures. Differences between 2 and 10 g/L substrate were generally not significant. Variability in substrate utilization efficiency was a result of compounding variability across multiple measurement systems. Variability associated with biomass growth, lipid and starch measurement, and substrate utilization measurements all contributed to variability in efficiency.

## Discussion

### The effects of bacterial contamination

It was initially hypothesized that the presence of a competitive organism such as *E. coli* would slow algae productivity due to resource competition, particularly for the carbon substrate. High substrate concentrations were expected to lead to cultures whose biomass was dominated by *E. coli* despite the significant initial cell density advantage afforded to *C. minutissima*.

The results suggest that neither hypothesis was correct: the co-culture had up to 592% greater algal biomass productivity than the axenic algal culture under 10 g/L substrate conditions. It also had greater lipid and starch production and could consume more of the available substrate than the axenic cultures at the 10 g/L substrate concentration. It appears that the two organisms exhibit something closer to a mutualistic relationship than a competitive one. Furthermore, substrate uptake by the co-culture appeared to be more than the sum of substrate uptake by each organism cultured alone. *E. coli* may also have benefited from the relationship as shown by an increased viable cell fraction in co-cultures compared to axenic cultures.

A number of hypotheses exist for algae-bacteria symbiosis. One hypothesis is that bacteria secrete hormones or co-factors that enhance algal growth. This was observed by de-Bashan et al. in which *Azospirillium* secreted IAA, thereby promoting growth in *Chlorella vulgaris* [14]. Likewise, Bajguz et al. showed that other phytohormones such as indole-3 propionic and indole-3 butyric acid had similar growth-promoting effects on autotrophic *C. vulgaris* cultures, particularly when supplied with brassinosteroids [34]. In addition to hormones, bacteria can synthesize vitamins that may confer growth advantages to algae. Croft et al. demonstrated that many algae species exhibit vitamin B_12_ auxotrophy, due largely to B_12_ dependence of methionine synthase [35]. They demonstrated that bacteria can supply algae with vitamin B_12_ in exchange for photosynthetic products. Moreover, they showed that *Chlamydomonas reinhardtii* contains two methionine synthase genes – one with vitamin B_12_ dependence and one without. The B_12_-dependent form has higher catalytic efficiency and is preferentially used when exogenous B_12_ is present indicating that symbiosis can occur even when algae are capable of fully autotrophic growth.

It is possible that the symbiotic relationship between *C. minutissima* and *E. coli* is the result of phytohormone or co-factor secretion. Vitamin B_12_ is not known to be produced by wild-type *E. coli* [36] which must use active transport to obtain it from their environment [37]. Likewise, *E. coli* are not known to produce IAA which is typically synthesized by soil bacteria (such as *Azospirillum*) [38]. Although the specific mechanisms discussed by de-Bashan et al. and Croft et al. are unlikely explanations for symbiosis between *C. minutissima* and *E. coli*, co-factors that assist enzymes specifically involved with organic carbon uptake and central metabolism could play a role in the observed symbiosis. Co-factors such as thiamine, riboflavin, pantothenic acid, biotin, and folic acid are synthesized by *Chlorella* and play a role in central metabolism [39], [40]. However, the effect of supplementation of these co-factors has not been well studied in mixotrophic algae cultures.

Another possible symbiotic mechanism is complimentary nutrient uptake and product secretion by the two organisms. For example, carbon dioxide evolution by *E. coli* in exchange for oxygen from algae could enhance overall growth rates in co-cultures. Such a relationship has been widely discussed as it pertains to wastewater treatment [41], [42]. This scenario is most plausible in a mixotrophic environment (as opposed to autotrophic conditions) since sufficient organic material must be present for significant bacterial CO_2_ production. Likewise, other products of primary metabolism could play a role in the observed symbiosis including metabolites secreted into the media by *C. minutissima* and *E. coli*. The nature of the symbiotic relationship is the subject of ongoing investigation.

### Effects of substrate on biomass growth

Productivities achieved in axenic *C. minutissima* cultures were comparable to those achieved in studies using other *Chlorella* strains. Liang et al. [5] achieved 254 mg/L/day, 102 mg/L/day, and 87 mg/L/day growing *Chlorella vulgaris* on 1% glucose, glycerol, and acetate respectively compared to 99, 123, and 60 mg/L/day in this study. Wan et al. [6] achieved 56 mg/L/day with *Chlorella sorokiniana* using 10 g/L glucose. Axenic cultures' substrate uptake appeared to be inhibited after 1–2 grams of substrate were utilized. This in turn, is the likely explanation for the observed saturation growth kinetics. Graphs presented by Liang et al. also indicated saturation behavior in mixotrophic cultures but no indication was made as to the cause.

The fraction of biomass as *C. minutissima* in co-cultures was significantly higher at 10 g/L substrate than it was at 2 g/L substrate, contrary to our hypothesis. *E. coli* biomass density, whether grown on its own or in co-cultures with *C. minutissima*, appeared to not exceed ∼0.5 g/L just as viable cell counts appeared to plateau at ∼10^9^ CFU/ml even though the carbon substrate was not exhausted. Monod identified three factors that can limit bacterial growth: 1) exhaustion of nutrients, 2) accumulation of metabolic products, and 3) changes in pH or ion equilibrium [43]. The plateau in *E. coli* growth could be related to oxygen limitation in spite of the presence of photosynthetic algae in co-cultures, particularly during the dark period. Lee et al. found that dissolved oxygen levels in mixotrophic *Chlorella* cultures could drop as low as 50% of saturation, even in the presence of light [44]. Products of *E. coli* anaerobic metabolism were not detected and were unlikely to play an inhibiting role, however, significant changes in pH over the culture period (despite adjustment) likely inhibited *E. coli* growth. Glass et al. showed that *E. coli* growth was inhibited below pH 6 and above pH 9, and complete inactivation was achieved at pH 4 [45]. In contrast, *C. minutissima* did not appear to have this limitation when grown in co-culture with *E. coli*. Once the maximum *E. coli* level was reached, additional culture growth appeared to come exclusively from *C. minutissima.* The majority of co-cultures provided with 10 g/L substrate were still growing at the end of the five day period suggesting that they still had not reached a biomass density limit. This process was likely truncated due to substrate limitation in the 2 g/L cultures and may explain why algae constituted a greater fraction of biomass in 10 g/L cultures (94% with glucose) than 2 g/L cultures (53% with glucose).

The relatively low efficiency of substrate utilization observed in this study (13–40%) indicates that there is room for improvement in terms of optimizing mixotrophic cultures. For reference, anaerobic fermentation of glucose to ethanol regularly yields a conversion efficiency, based on enthalpy of combustion, approaching 70% (1.43 mol ethanol per mol glucose) [46]. Mixotrophic algae cultures ideally operate as aerobic systems in which intracellular biofuel precursors are produced as opposed to a secreted anaerobic product. Acid formation by *C. minutissima* appeared to correlate with uptake of glucose and glycerol but common anaerobic metabolites were not identified in the media. Future work is required to identify products secreted by *C. minutissima* that are specifically linked to substrate consumption. Axenic *E. coli* cultures secreted detectable formic and acetic acids into the media which are common bi-products of *E. coli* fermentation [47], however, these products were not detected in the co-cultures in spite of the pH decline observed. One explanation is that the two organisms were exchanging metabolites that were secreted into the media (e.g. *C. minutissima* consumed acetate secreted by *E. coli* when grown on glucose) but this explanation requires further investigation.

### Effect of substrate and kanamycin on biomass composition

Cultures grown on glucose and glycerol had higher starch levels than those grown on acetate. Glucose and glycerol enter the glycolytic pathway upon phosphorylation by hexokinase and glycerol kinase respectively whereas acetate enters central metabolism as acetyl-CoA [27]. Synthesis of glycolytic intermediates from acetyl-CoA can be accomplished through OAA production via the glyoxylate cycle followed by gluconeogenesis [27]. The gluconeogenic pathway consumes net ATP, which could explain the low starch content in acetate cultures. For axenic *C. minutissima*, autotrophic cultures were found to have higher total lipid content than mixotrophic cultures. This is consistent with the findings of Chen and Johns who observed decreased crude lipid in *Chlorella* when glucose concentration was increased but the cause was unclear [48]. Interestingly, they also observed increased fatty acid content under higher glucose concentrations.

In co-cultures, the substrate concentration appeared to affect the lipid content modestly, and starch content significantly. Specifically, the 10 g/L substrate condition led to large and statistically significant increases in starch content. The likely explanation is that *C. minutissima* was primarily storing the excess substrate as starch rather than lipids. This effect was suppressed in axenic *C. minutissima* cultures but the effect was likely due to kanamycin rather than any effect of *E. coli*. Axenic *C. minutissima* grown on glucose in the absence of kanamycin had starch levels comparable to those found in the co-cultures.

Kanamycin is an inhibitor of the 30 S ribosomal unit found in prokaryotes. Any effect it has on eukaryotic algae is likely to be limited to protein synthesis in the chloroplast. Little research has been conducted on protein synthesis in chloroplasts of *C. minutissima* but synthesis of chloroplast proteins are often shared between the nucleus and chloroplast nucleoids in plants and algae [49]. Theoretically, genes associated with starch synthesis and degradation could be housed in the nucleoids whereas those associated with lipids could be housed in the nucleus. Commercial algae production is unlikely to employ antibiotics unless very high value products are produced. For this reason, the effect observed here has little commercial relevance.

### Implications for large-scale cultivation

Until recently, algae have been considered primarily as a tertiary wastewater treatment process in which the majority of organic carbon is consumed in upstream processes [11], [50], [51]. However, recent success with cultivating algae monocultures on primary wastewaters has led some researchers to conclude that algae can be used for secondary wastewater treatment [52]. *Chlorella* in particular has been identified as a promising candidate for organic carbon removal due to its mixotrophic capabilities [52]. However, these studies overlook the potentially significant impact that heterotrophs would play in such systems. Thus, improved understanding of algal-bacterial interaction in mixotrophic systems is required.

Although the present findings are not representative of the complexity found in a wastewater treatment process, they do shed light on important organism interactions that can take place in organic-rich waters. These results demonstrate that *Chlorella* can compete (and thrive) with a heterotrophic organism, even in nutrient-rich waters. Moreover, simultaneous cultivation of multiple organisms can lead to more productive outcomes (e.g. greater biofuel production and organic carbon removal from water as in the present study) than cultivation of algae monocultures, which have comprised the majority of academic research to date [5], [9]–[11]. Other researchers have shown the benefits of co-culturing multiple photosynthetic organisms for improved removal of nitrogen, phosphorus, [53] and organic acids [54]. The results of these two studies highlighted the importance of ecological niche in mixed cultures where organisms preferentially consumed different substrates [54] and occupied different culture zones within the reactors [53]. Additional research into co-cultures on various algal and bacterial species is required in order to broaden understanding of microbial interactions in algal cultures.

Despite the productivity benefits of algal-bacterial co-cultures, challenges such as flocculation and bio-fouling remain. Because the fouling in the present study did not begin until culture growth had reached the late-log phase, it is unlikely that it played a significant role in the elevated productivity of co-cultures in the first few days of growth. However, it may have affected cell growth and composition during the final days of the culture period. Discussion of bacterial flocculation in the literature suggests that bacteria can cause algae to flocculate [55], [56], indicating that this phenomenon may not be limited to the present study. Raszka et al. discuss the role of polymers secreted by certain bacteria in the flocculation process [55]. Although bio-fouling due to coagulation of co-cultures proved challenging in air-lift reactors, coagulation may provide benefits in large-scale systems. There is great interest in using biological flocculation as an inexpensive harvesting system for microalgal cultures [57]. Salim et al. point out that biological flocculating systems can eliminate the need for costly separation processes required to recover chemical flocculants [57]. The challenge, however, is managing the process such that both high productivity and high harvesting efficiency can be achieved.

## Conclusions

The results of the present study indicate that co-culture of the green algae *C. minutissima* with *E. coli* under mixotrophic conditions can enhance algal productivity. Although the co-culture represents a simplification of a “real” algae cultivation system, the results provide insight into how algae can respond to a heterotrophic organism. Future work is necessary to further elucidate the complex interactions between *E. coli* and *C. minutissima*, however. Further research on co-cultures of different algal and bacterial species can provide insight into the types of species interactions that could take place in scaled-up systems. These results, in turn, should lead to improved management practices for mixotrophic algae cultivation.

## Supporting Information

Figure S1 **Growth curves of axenic** ***C. minutissima*** **on glucose.** Bars are standard deviations.
(TIF)

Figure S2 **Growth curves of axenic** ***C. minutissima*** **on glycerol.** Bars are standard deviations.
(TIF)

Figure S3 **pH of media over time in cultures of axenic** ***C. minutissima*** **grown on glucose.**
(TIF)

Figure S4 **pH of media over time in cultures of axenic** ***C. minutissima*** **grown on acetate.**
(TIF)

Figure S5 **Viable** ***E. coli*** **in co-culture grown on glucose.**
(TIF)

Figure S6 **Viable** ***E. coli*** **in co-culture grown on glycerol.**
(TIF)

Figure S7 **Viable** ***E. coli*** **in co-culture grown on acetate.**
(TIF)

Figure S8 **Growth curves of axenic** ***E. coli*** **on acetate.** Bars are standard deviations. Large variation was observed at 2 g/L acetate at 120 hours since each culture appeared to enter exponential growth at a different time.
(TIF)

Figure S9 **Growth curves of axenic** ***E. coli*** **on glucose.** Bars are standard deviations. At 96 hours, 0.2 g/L glucose remained in cultures originally supplied with 2 g/L glucose.
(TIF)

Figure S10 **pH of media over time in cultures of axenic** ***E. coli*** **grown on glucose.** pH was adjusted every 24 hours.
(TIF)

Figure S11 **Growth curves of axenic** ***E. coli*** **on glycerol.** Bars are standard deviations.
(TIF)
