# Supplementary figures and images for: Effects of Escherichia coli on Mixotrophic Growth of Chlorella minutissima and Production of Biofuel Precursors
Source: PLoS One. 2014 May 7;9(5):e96807. doi: 10.1371/journal.pone.0096807 (PMC4013066; doi:10.1371/journal.pone.0096807)

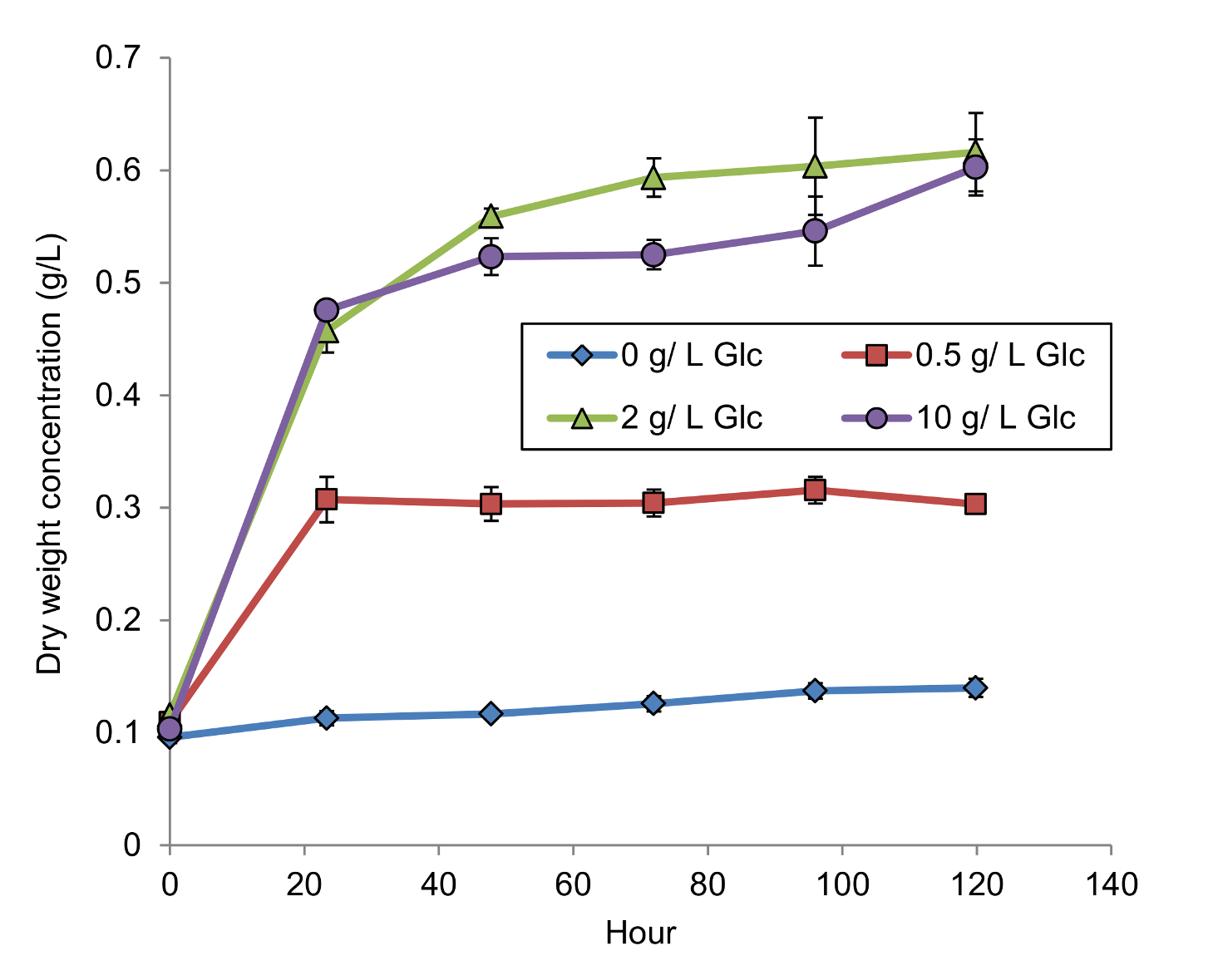

Supplement: Figure S1 — Growth curves of axenic C. minutissima on glucose. Bars are standard deviations. (TIF) [file pone.0096807.s001.tif]

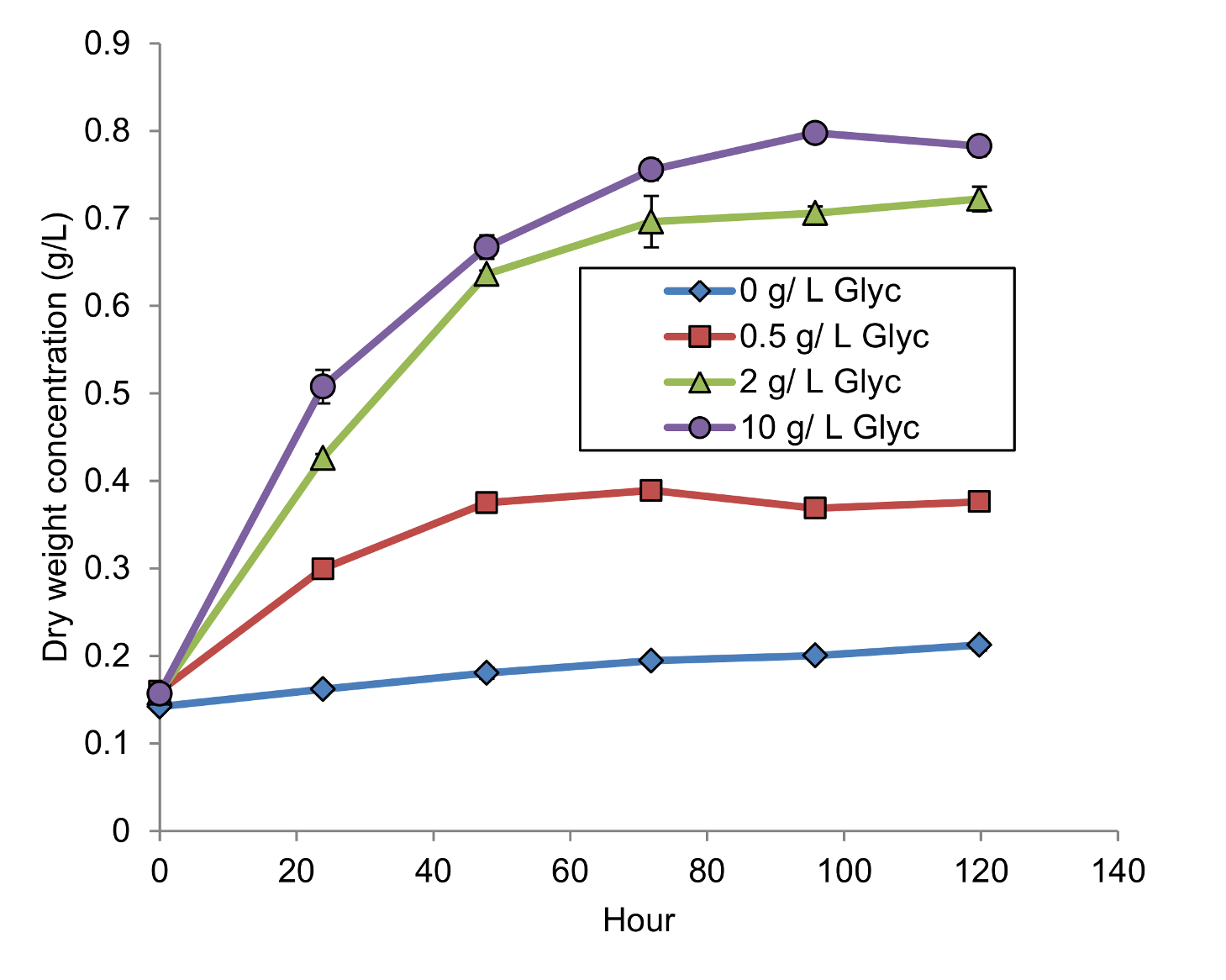

Supplement: Figure S2 — Growth curves of axenic C. minutissima on glycerol. Bars are standard deviations. (TIF) [file pone.0096807.s002.tif]

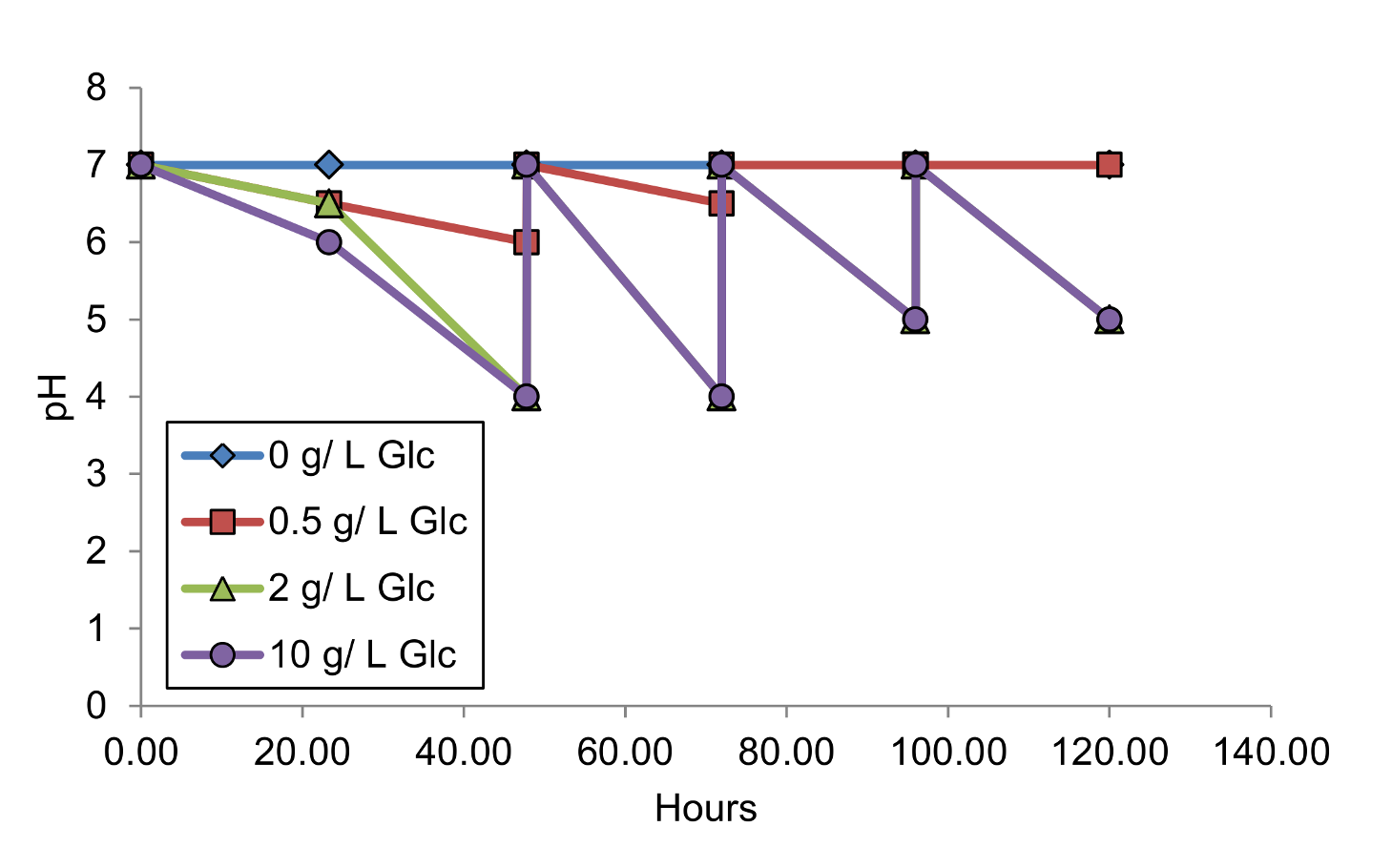

Supplement: Figure S3 — pH of media over time in cultures of axenic C. minutissima grown on glucose. (TIF) [file pone.0096807.s003.tif]

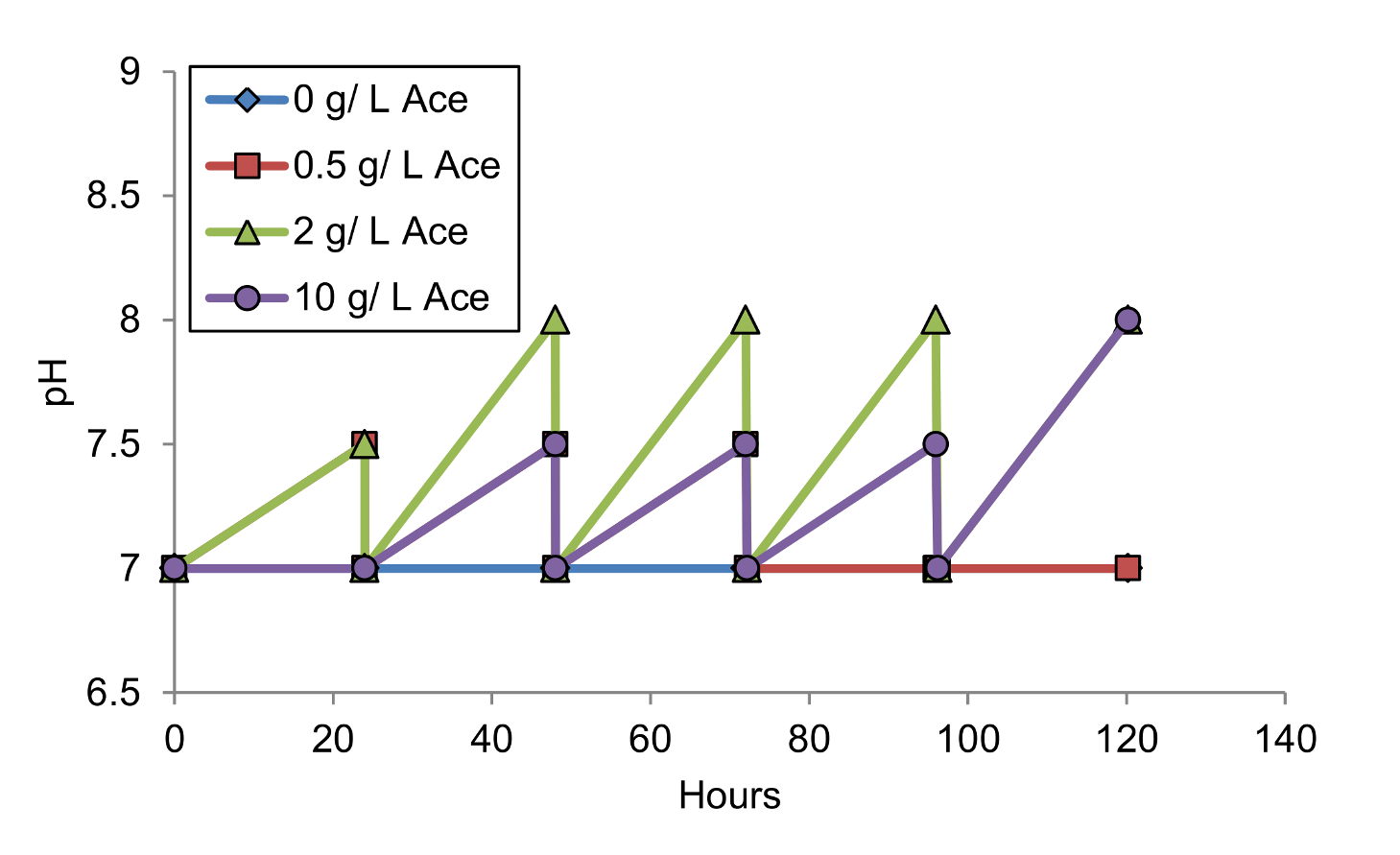

Supplement: Figure S4 — pH of media over time in cultures of axenic C. minutissima grown on acetate. (TIF) [file pone.0096807.s004.tif]

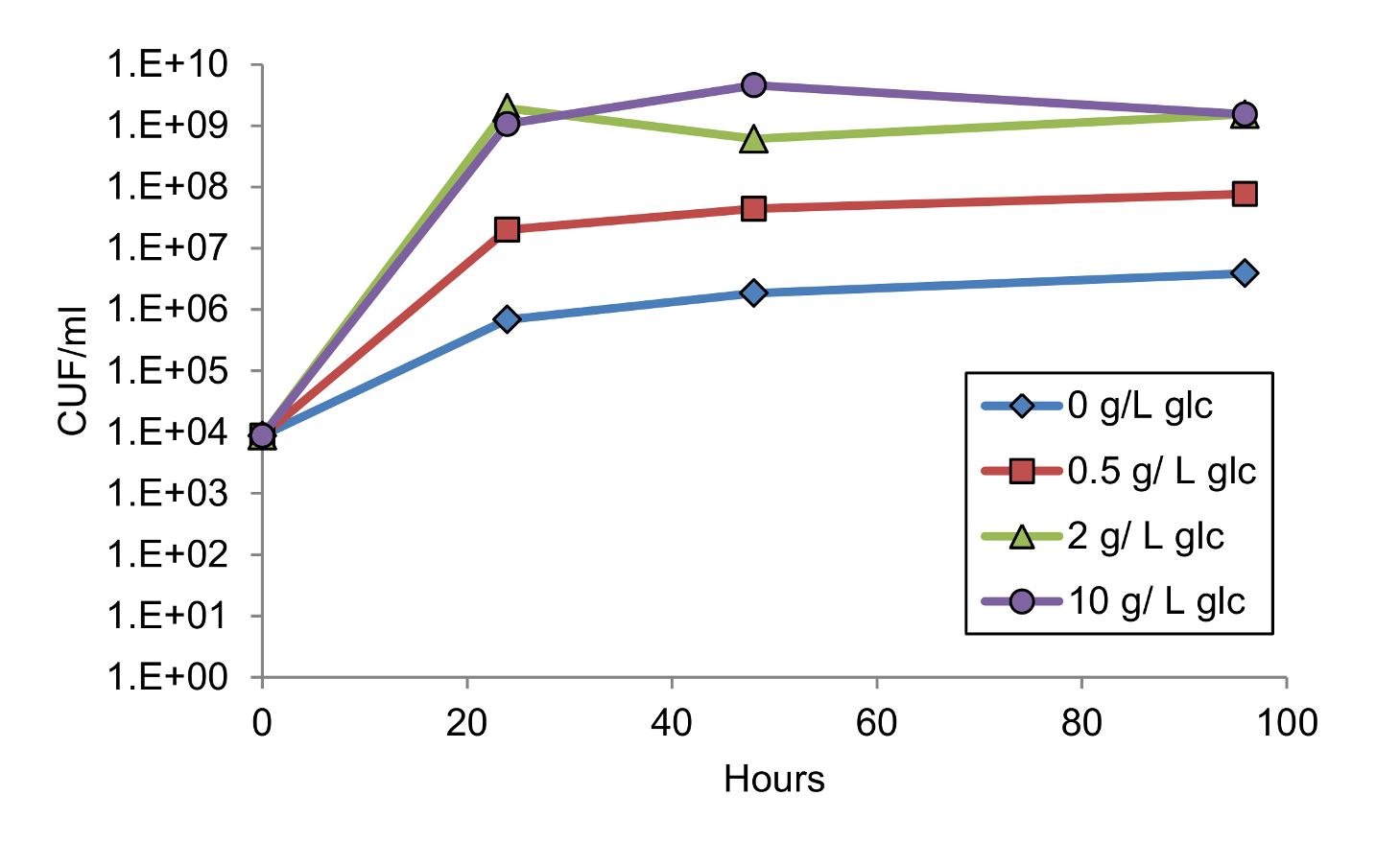

Supplement: Figure S5 — Viable E. coli in co-culture grown on glucose. (TIF) [file pone.0096807.s005.tif]

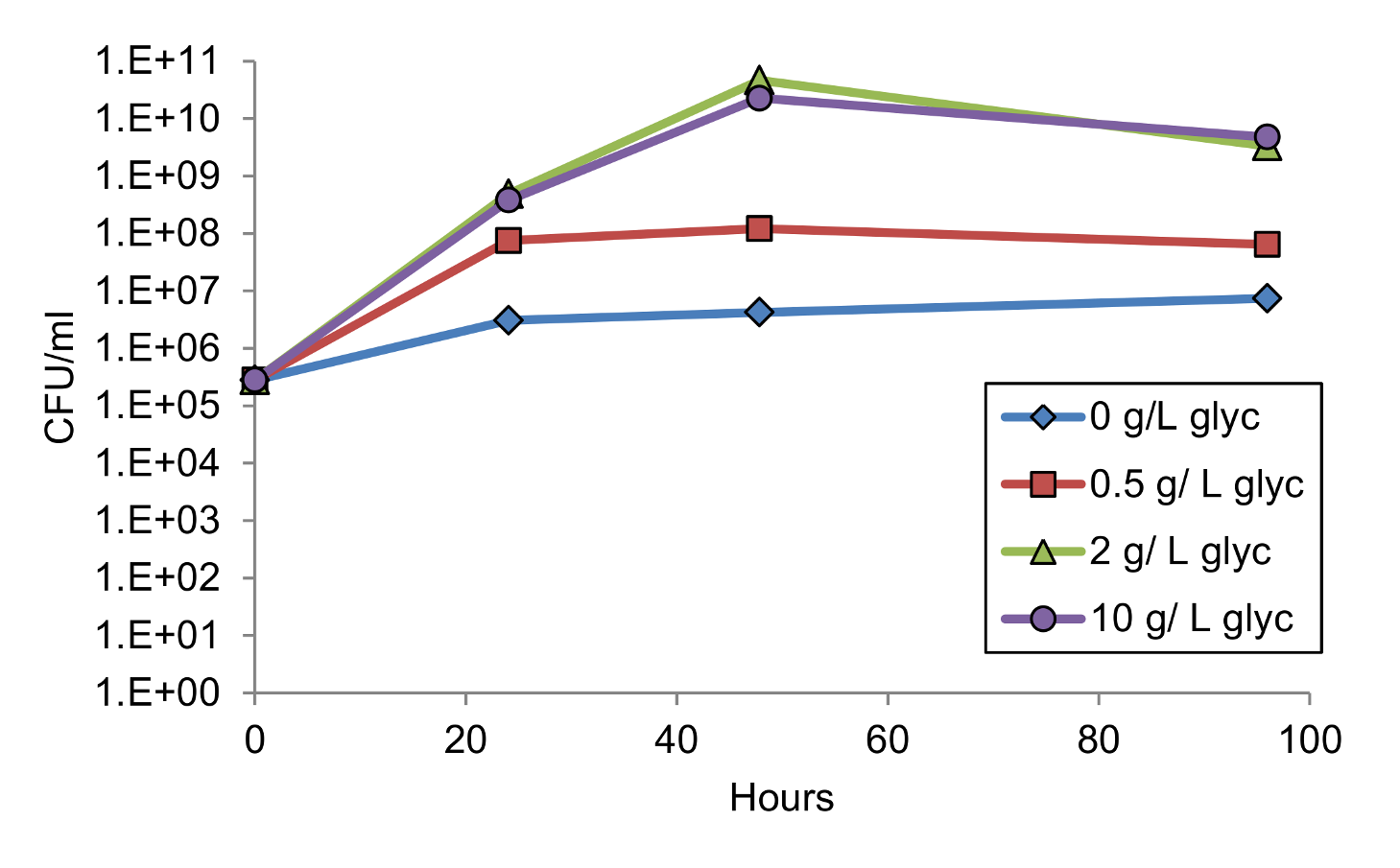

Supplement: Figure S6 — Viable E. coli in co-culture grown on glycerol. (TIF) [file pone.0096807.s006.tif]

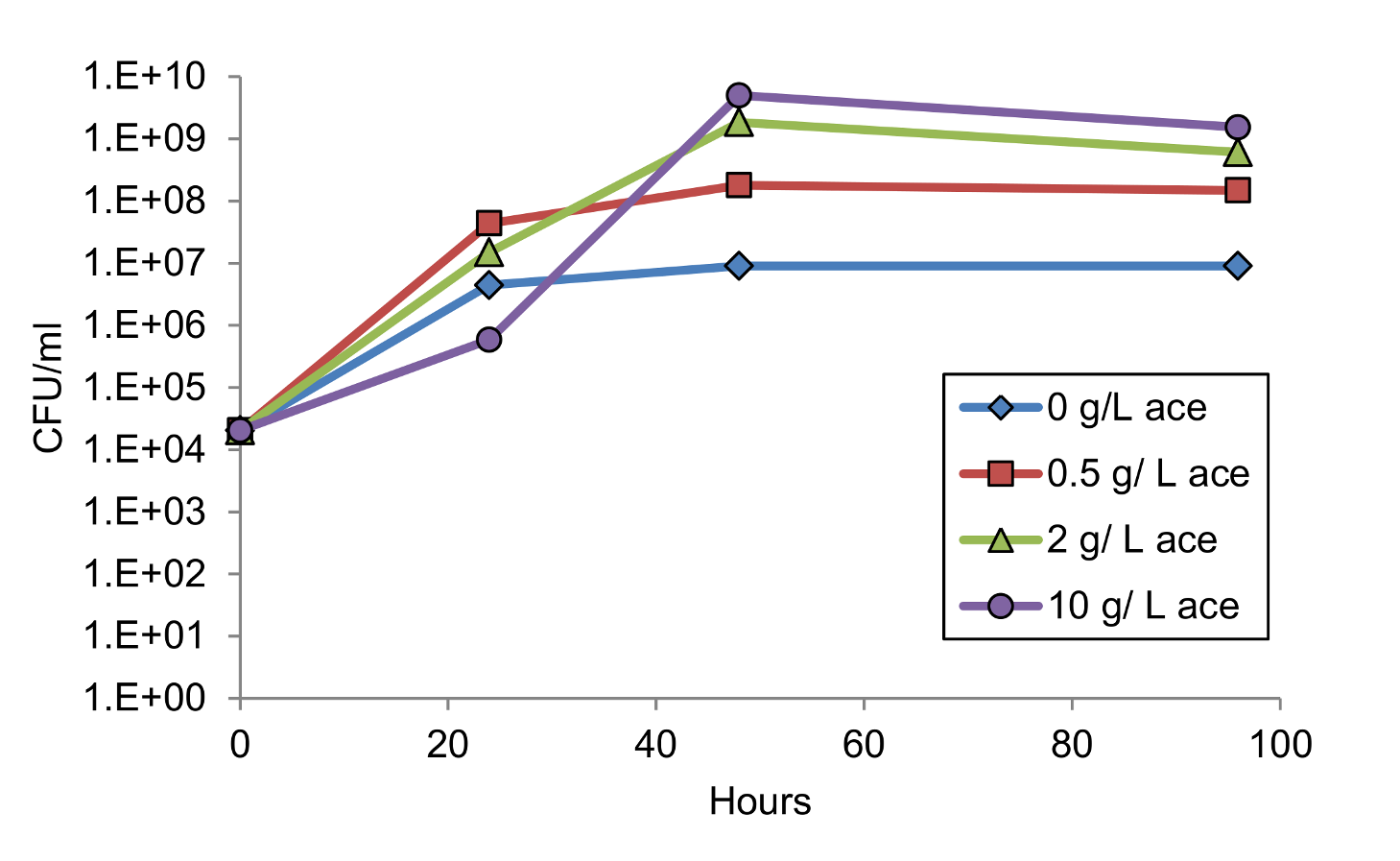

Supplement: Figure S7 — Viable E. coli in co-culture grown on acetate. (TIF) [file pone.0096807.s007.tif]

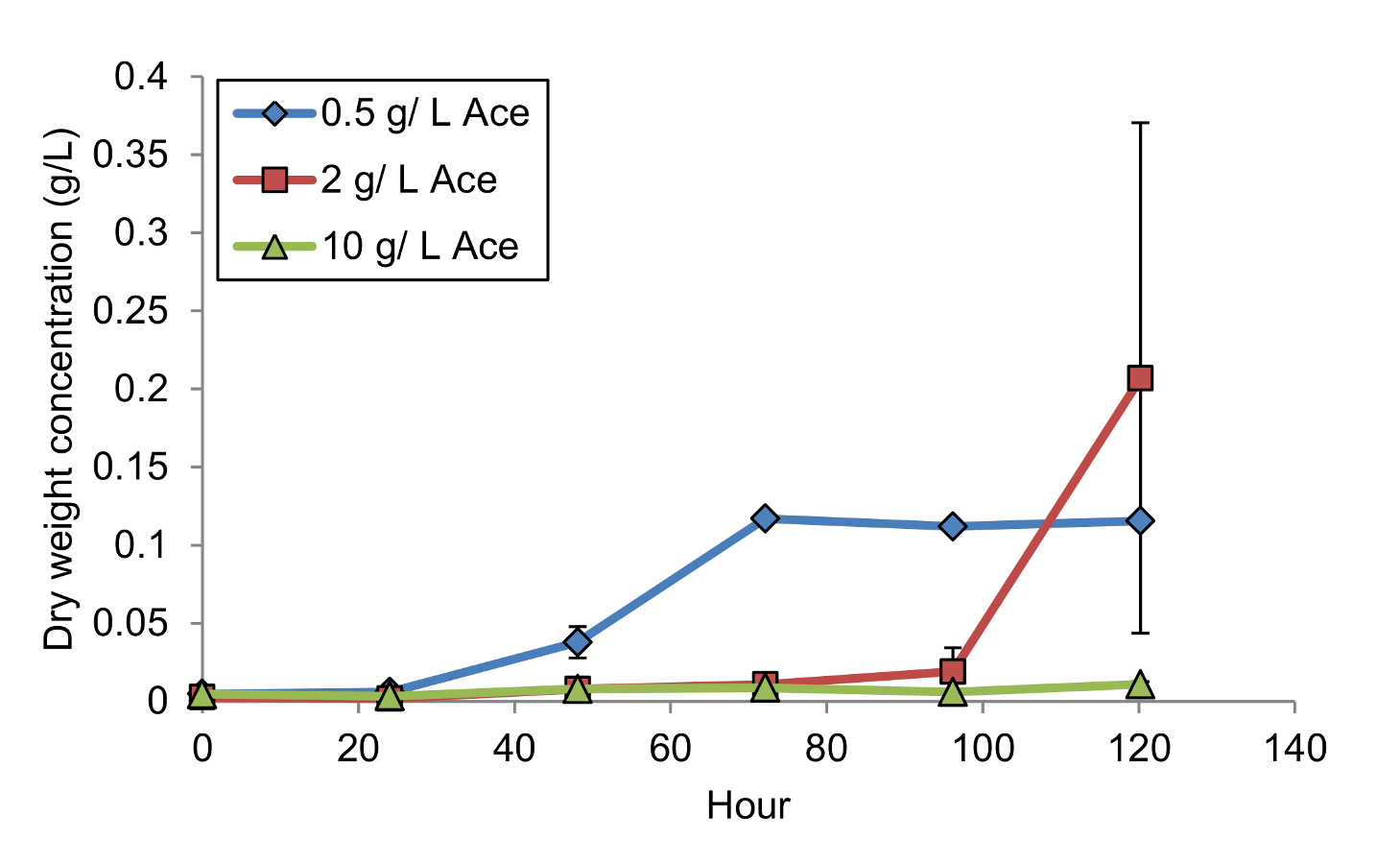

Supplement: Figure S8 — Growth curves of axenic E. coli on acetate. Bars are standard deviations. Large variation was observed at 2 g/L acetate at 120 hours since each culture appeared to enter exponential growth at a different time. (TIF) [file pone.0096807.s008.tif]

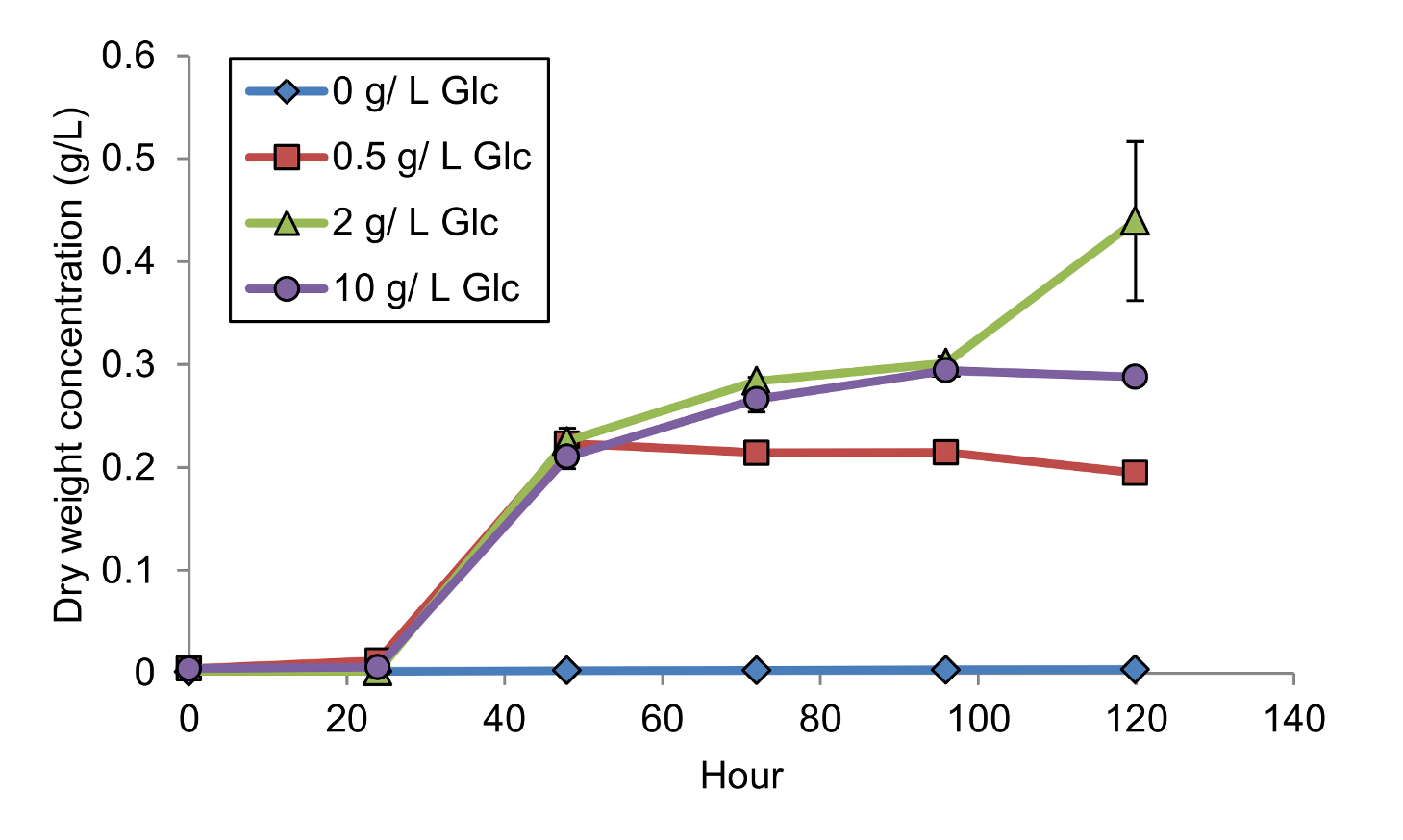

Supplement: Figure S9 — Growth curves of axenic E. coli on glucose. Bars are standard deviations. At 96 hours, 0.2 g/L glucose remained in cultures originally supplied with 2 g/L glucose. (TIF) [file pone.0096807.s009.tif]

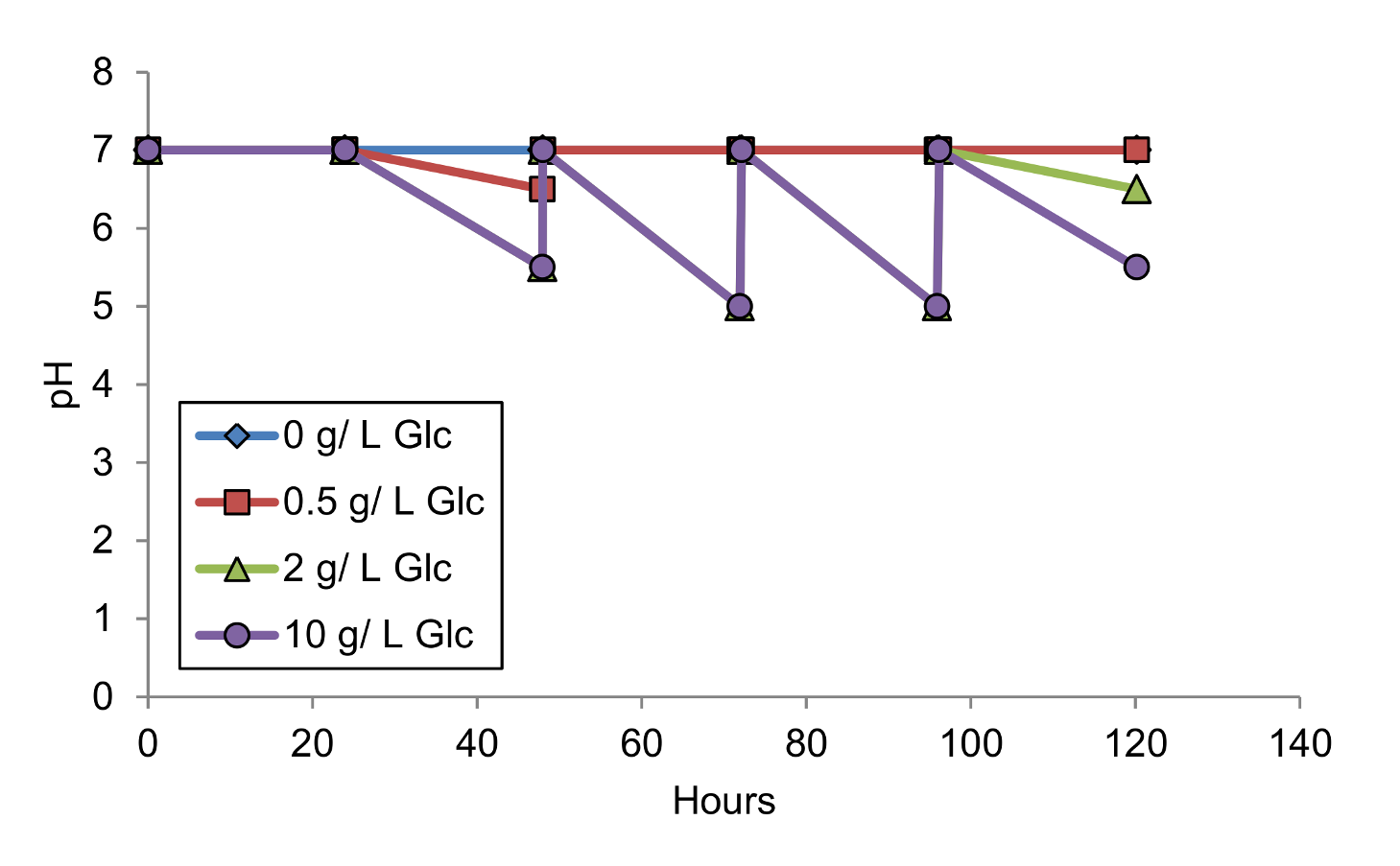

Supplement: Figure S10 — pH of media over time in cultures of axenic E. coli grown on glucose. pH was adjusted every 24 hours. (TIF) [file pone.0096807.s010.tif]

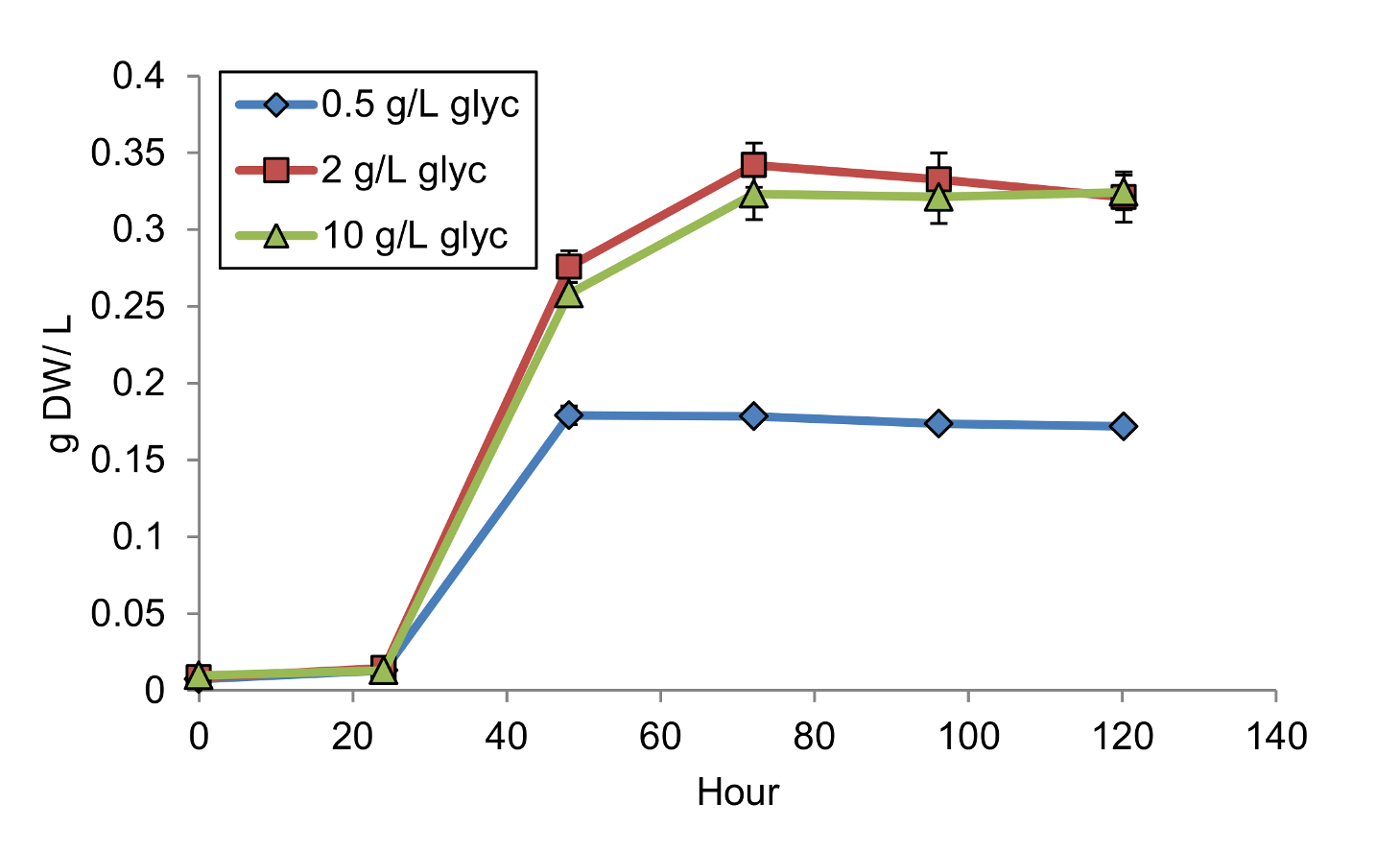

Supplement: Figure S11 — Growth curves of axenic E. coli on glycerol. Bars are standard deviations. (TIF) [file pone.0096807.s011.tif]
